# Synthesis of TiO_2_-Cu^2+^/CuI Nanocomposites and Evaluation of Antifungal and Cytotoxic Activity

**DOI:** 10.3390/nano13131900

**Published:** 2023-06-21

**Authors:** Rafael Hernandez, Arturo Jimenez-Chávez, Andrea De Vizcaya, Juan Antonio Lozano-Alvarez, Karen Esquivel, Iliana E. Medina-Ramírez

**Affiliations:** 1 Department of Chemistry, Universidad Autónoma de Aguascalientes, Av. Universidad 940, Aguascalientes 20100, Mexico; rafa.hdz2109@outlook.com; 2 Departamento de Toxicología, Centro de Investigación y Estudios Avanzados del IPN, Ciudad de Mexico 07360, Mexico; 3 Department of Environmental and Occupational Health, Program in Public Health, Susan and Henry Samueli College of Health Sciences, University of California, Irvine, CA 92697, USA; 4 Department of Biochemical Engineering, Universidad Autónoma de Aguascalientes, Av. Universidad 940, Aguascalientes 20100, Mexico; 5 División de Investigación y Posgrado, Facultad de Ingeniería, Universidad Autónoma de Querétaro, Cerro de las Campanas, Santiago de Querétaro 76010, Mexico

**Keywords:** antifungal, composites, copper(I) iodide, cytotoxicity, titanium dioxide

## Abstract

Fungal infections have become a significant public health concern due to their increasing recurrence and harmful effects on plants, animals, and humans. Opportunistic pathogens (*among others from the genera Candida and Aspergillus*) can be present in indoor air, becoming a risk for people with suppressed immune systems. Engineered nanomaterials are novel alternatives to traditional antifungal therapy. In this work, copper(I) iodide (CuI) and a copper-doped titanium dioxide—copper(I) iodide (TiO_2_-Cu^2+^/CuI) composite nanomaterials (NMs)—were synthesized and tested as antifungal agents. The materials were synthesized using sol-gel (TiO_2_-Cu^2+^) and co-precipitation (CuI) techniques. The resulting colloids were evaluated as antifungal agents against *Candida parapsilosis* and *Aspergillus niger* strains. The NMs were characterized by XRD, HRTEM, AFM, and DLS to evaluate their physicochemical properties. The NMs present a high size dispersion and different geometrical shapes of agglomerates. The antifungal capacity of the NMs by the minimum inhibitory concentration (MIC) and minimum fungicidal concentration (MFC) was below 15 µg/mL against *Candida parapsilosis* and below 600 µg/mL against *Aspergillus niger* for both NMs. Holotomography microscopy showed that the NMs could penetrate cell membranes causing cell death through its rupture and reactive oxygen species (ROS) production. Cytotoxicity tests showed that NMs could be safe to use at low concentrations. The synthesized nanomaterials could be potential antifungal agents for biomedical or environmental applications.

## 1. Introduction

Health care related to the increase in fungal infections and fungal contamination is currently a global concern. Medical advances and developments in treating cancer, immunosuppression, and immune dysfunction have improved and lengthened human life. However, these treatments have also increased susceptibility to fungal infections, which can lead to systemic fungal diseases and even become life-threatening conditions [1,2]. At the same time, the extensive use of antifungal and antibacterial products in agriculture and medicine has altered the global microbiome, leading to drug-resistant fungal infections in plants, animals, and human beings [1,3].

A wide variety of fungal infections can affect human beings, from superficial skin infections to systemic infections invading internal organs [4]. Most invasive fungal infections worldwide are caused by *Candida*, *Cryptococcus*, *Aspergillus,* and *Pneumocystis* species [5,6]. Generally, these infections affect immunocompromised hosts and patients who are apparently healthy and immunocompetent but have asymptomatic conditions that might alter immune function [7]. Hence, the treatment of such infections represents a global health burden.

The current available antifungal drugs should be potent and effective, which can be grouped into four main classes (polyenes, azoles, allylamines, and echinocandins). However, as with any therapy, these drugs have drawbacks regarding their activity spectrum, drug–drug interactions, pharmacokinetics and pharmacodynamics, resistance mechanism, and the compounds’ toxicity [4,5]. Therefore, an increasing interest is in developing novel antifungal compounds or enhancing those currently available.

Among current developments, engineered nanomaterials and nanotechnology have gained the scientific community’s attention due to their potential therapeutic applications. Numerous studies have assessed the potential toxicity of nanoparticles on several biological systems, including bacteria, fungi, yeasts, and even whole organisms [8]. Some of the most promising nanoparticles (NPs) utilized in recent research works are semiconductor metal oxides such as titanium dioxide (TiO_2_) doped with metal ions such as copper (Cu), iron (Fe), and cerium (Ce) [9,10,11]. For instance, Cu-doped TiO_2_ nanoparticles have been reported to have antimicrobial activity against *Phytophthora palmivora*, *Escherichia coli*, and *Staphylococcus aureus* [12,13,14]. Furthermore, copper-based nanomaterials such as copper(I) iodide (CuI) have attracted the interest of many researchers due to their exceptional properties and diverse fields of application [15,16]. Antibacterial and antifungal properties of CuI have been previously reported against different fungi (*Sporothrix schenckii*, *Candida albicans*) and bacteria strains (*Staphylococcus aureus, Escherichia coli, Bacillus subtilis, Pseudomonas aeruginosa*), which indicates the reliable use of the material as a disinfectant agent due to its high performance [7,17]. Nevertheless, the use of nanocomposites, such as TiO_2_-Cu^2+^/CuI, to reduce its toxicity and increase its bioactivity has not been fully explored.

The biological pollution of indoor air is a risk factor in the spread of several infectious diseases [18,19]. Numerous publications discuss the problems encountered by healthcare personnel due to the presence of different microorganisms in intensive care units (ICUs) or operating rooms. More than viruses or bacteria, airborne fungi (or spores) enter a building that uses natural ventilation. *Aspergillus* sp. is considered a potentially life-threatening airborne contaminant for immunocompromised individuals. Exposure to *Aspergillus* can result in different clinical manifestations, from allergic to invasive disease, which depends on the patient’s immune system and structural lung disease [20]. *Aspergillus niger* is a representative airborne microorganism since its spores are present in indoor environments and air conditioner filters; these spores show resistance to any environmental stress conditions becoming omnipresent. Furthermore, Candida species cause approximately 80% of invasive fungal infections in ICUs [2,21]. These infections are linked with a high risk of death in patients with septic shock with delayed antifungal therapy. In this study, we use unicellular (*C. parapsilosis*) and multicellular (*A. niger*) fungal species as model organisms to evaluate the antifungal activity of TiO_2_-Cu^2+^/CuI NMs. These fungi are associated with numerous health effects on humans, highlighting the importance of their removal from indoor air.

In this work, we aim to synthesize composite NMs using robust, reproducible green routes. In this regard, a sol-gel procedure allows the incorporation of Cu^2+^ dopant into the TiO_2_ lattice [22]. Furthermore, the synthesis of CuI NMs undergoes using a controlled precipitation technique. We use a biopolymer as a surfactant, rendering stable colloidal suspensions [17]. Since CuI is prone to oxidation and dissolution, the composite increases its stability (retards oxidation of Cu^+^ ion and decreases CuI solubility). The composition of the composite under study (TiO_2_, Cu^+^, Cu^2+^, I^−^) offers numerous possibilities to inhibit fungal growth. For instance, copper and copper-containing compounds are known to be efficient wide-spectrum antimicrobial agents. Copper (I) compounds show better antibacterial and antiviral activity compared to Cu(II) compounds [23]. In addition, CuI and TiO_2_ cause ROS-mediated cellular damage in numerous microorganisms [7,24,25].

Although the main application of the TiO_2_-Cu^2+^/CuI under study is for air purification, we consider it an ethically responsible action to evaluate NM toxicity in the scenario of their accidental release. Typically, NMs in the air can travel large distances by Brownian diffusion and become air pollutants that can be inhaled by human beings [26]. Depending on the composition and physicochemical properties of NMs, exposure to these NMs could result in diverse adverse effects [27]. For example, a study by Bai et al. shows the health impacts of Cu NPs after their intranasal instillation. The Cu NMs can enter the brain and induce damage to the central nervous system [28]. Moreover, air pollution (in particular, exposure to ultra-fine particles) is responsible for an increase in morbidity and mortality from respiratory (asthma, obstructive pulmonary disease, cancer, pulmonary fibrosis) and cardiovascular diseases [29].

Earlier reports point out the efficient and robust catalytic activity of CuI/TiO_2_ composites to convert aryl acetylenes to α-ketoaryl amides [30]. Recent studies reveal the enhanced activity of CuI/TiO_2_ composites for environmental applications: ultraviolet photodetection [31], the electro-oxidation of methanol [32], and p-type transparent conduction [33]. To our knowledge, there are no studies regarding the synthesis, property evaluation, and application of TiO_2_-Cu^2+^/CuI NMs. In this work, it was proved that TiO_2_-Cu^2+^/CuI NMs exhibit suitable properties for the development of sustainable environmental remediation technologies. Previously, the efficiency of TiO_2_-Cu^2+^ NMs was demonstrated for water disinfection [22]. We also investigated the antifungal activity of CuI NMs [17]. In this study, we are interested in fabricating a photocatalytic material with enhanced photocatalytic activity for air purification. Improving indoor air quality (IAQ) is a suitable strategy to decrease the probability of fungal infection transmission and improve the health wellness of susceptible individuals (children, elderly, or immunocompromised individuals).

Herein, the synthesis, characterization, and antifungal activity of CuI and the TiO_2_-Cu^2+^/CuI nanocomposite is reported against *Candida parapsilosis* and *Aspergillus niger* as model organisms. The synthesized nanocomposites have a high potential to become candidates for biomedical applications or the development of environmental remediation (air treatments) technologies. Advanced microscopic techniques (atomic force microscopy and holotomography microscopy) were used to study the interaction of nanocomposites with fungal strains and human bronchial epithelial (BEAS-2B) cells. In addition, classical toxicity assays prove the biocompatibility of the materials with human bronchial epithelial cells (BEAS-2B), demonstrating the safe use of the nanomaterials.

## 2. Materials and Methods

### 2.1. Materials and Reagents

Titanium isopropoxide (Sigma-Aldrich, St. Louis, MO, USA, 97%), Arabic gum (Golden Bell, Zapopan, México), copper (II) sulfate pentahydrate (Karal, León, México), potassium iodide (J.T. Baker, Cuautitlán Izcalli, México), acetic acid (J.T. Baker), and hydrazine hydrate (Sigma-Aldrich, St. Louis, MO, USA, 60%) were all analytical grade and were used as received. The microbiological media Sabouraud dextrose agar (BD Bioxon, Cuautitlán Izcalli, México), potato dextrose agar (BD Bioxon, Cuautitlán Izcalli, México), and potato dextrose broth (BD Difco^TM^, Cuautitlán Izcalli, México) were used according to the suppliers’ instructions.

### 2.2. Synthesis of Nanostructured Materials (NMs)

The TiO_2_-Cu^2+^ (1%) synthesis was carried out using a previously published sol-gel process with slight changes [22]. Briefly, to obtain 1% (atomic Cu/Ti) doping of titanium dioxide, copper (II) sulfate was added to 10 mL of glacial acetic acid and magnetically stirred until complete dissolution. Afterward, titanium isopropoxide was added to the copper solution while stirring and mixed fully for 10 min. Finally, 30 mL of a previously prepared 3 wt.% Arabic gum solution was added dropwise, and the final solution was stirred at room temperature for 18 h. The obtained gel was dried at 70 °C and calcined at 350 °C for 3 h. The resulting powder was ground and stored for further use.

The copper(I) iodide materials were synthesized by a modification of the method reported by Pramanik et al. [24] and Martínez-Montelongo et al. [17]. Briefly, 45 millimolar potassium iodide was added to 45 millimolar copper (II) sulfate with continuous stirring in 35 mL of 3 wt.% Arabic gum as stabilizing agent. Next, the reaction solution was placed in an ice bath until the temperature reached 4 °C. Afterwards, 90 µL of hydrazine hydrate was added dropwise, and the reaction was carried out for 1 h; subsequently, the temperature was increased to 60 °C for 1 h. Finally, the resulting white solution was stirred for 18 h.

The synthesis of the TiO_2_-Cu^2+^/CuI composite underwent using a weight TiO_2_: CuI ratio of 1:2. In brief, the previously synthesized TiO_2_-Cu^2+^ powders were added to 35 mL of a 3 wt.% Arabic gum solution and sonicated for 30 min to ensure proper dispersion of the nanoparticles. The following procedure was the same as the previously described CuI synthesis. The TiO_2_-Cu^2+^/CuI and CuI NMs were obtained as colloids and used as prepared by adjusting with Arabic gum solution to the desired concentration. 

### 2.3. Characterization of Nanomaterials

The synthesized nanomaterials (NMs) were characterized by transmission electron microscopy (TEM, JEOL ARM 200F operating at 200 kV) to analyze the morphology of the particles, and atomic force microscopy (AFM, Bruker, Dimension Edge with Scan Asyst) was carried out to assess the topography of the nanomaterials under study. For solid characterization, the nanomaterials were separated by centrifugation (8000 rpm, 10 min; OHAUS FC5816), washed with an ethanol/water mixture (50:50 *v*/*v*), and let dry in an oven at 60 °C before characterization. Thin films of CuI and TiO_2_-Cu^2+^/CuI were deposited on a glass substrate using the doctor blade technique [34]. The surface roughness and topography at the micro- and nanometer scales were analyzed using ScanAsyst equipment (Bruker, Dimension Edge with Scan Asyst). The samples were examined in scanasyst mode under an air atmosphere with SCANASYST-AIR tips (silicon tip on nitride lever; T: 650 nm; f_0_ = 70 kHz; k = 0.4 N/m). Images were recorded using height, adhesion, and peak force channels on square frames of 5 × 5 and 2.5 × 2.5 µm. The crystalline structure of the NMs was determined by means of X-ray diffraction (XRD) using a Siemens D5000 diffractometer with Co-Κ_α_ radiation (λ = 1.7903 Å) and a step size of 0.020°. In addition, the size and morphology of the agglomerates were studied by HRTEM in a JEOL ARM 200F operating at 200 kV. Selected-area diffraction patterns (SADP) were recorded to determine the crystalline phases present in the composite.

The CuI and TiO_2_-Cu^2+^/CuI were analyzed to examine their hydrodynamic diameter and Zeta potential. Both materials were characterized in water by preparing a 1 mg/mL suspension of NMs and sonicated for 15 min in a sonic bath; 1 mL of the suspension was taken as a sample. The hydrodynamic diameter was determined by dynamic light scattering analysis (DLS) and Zeta potential by laser Doppler microelectrophoresis (Zetasizer NanoCytotoxicityZS90 from Malvern Instruments Ltd., Malvern, UK).

### 2.4. Antifungal Activity of CuI-Based Nanostructured Materials

The antifungal activity of the nanomaterials was evaluated against *Candida parapsilosis* and *Aspergillus niger* and used as a reference to demonstrate the enhanced activity of the TiO_2_-Cu^2+^/CuI nanocomposite. The *Candida parapsilosis* strain used in this study was obtained from a clinical isolate, and the *Aspergillus niger* strain was isolated from a clinical air sample. Isolates were cultured in Sabouraud dextrose agar (SDA) and incubated at 35 °C for 48 h. Afterward, fungal biomass was collected with a sterile loop and placed in 1 mL of phosphate-buffered saline (PBS) to obtain a stock fungal suspension. An inoculum of *Candida parapsilosis* was resuspended in 3 mL of potato dextrose broth (PDB) and left to grow at 35 °C for 48 h in a shaking water bath at 60 rpm. Next, the fungal suspension was centrifuged at 3500 rpm for 5 min, decanted, and suspended in sterile distilled water. Finally, the suspension was counted before all experiments in a Neubauer chamber and diluted to 1 × 10^7^ cells/mL in sterile distilled water. In the case of the *Aspergillus niger* strain, the isolates were cultured in potato dextrose agar (PDA) and incubated at room temperature for seven days. Afterward, the fungal spores were collected by adding 5 mL of a 0.01 wt.% Tween 80 to the Petri dish, the recovered spore suspension was filtered and counted prior to experiments in a Neubauer chamber and diluted to 1 × 10^7^ spores/mL.

The interaction tests were carried out in a 2 mL volume in test tubes with a suspension containing 1 × 10^6^ cells/mL made by dilution from the stock fungal suspension. Before the interaction, the synthesized NMs colloids were adjusted at a fixed concentration (2000 µg/mL) in a 1.5% Arabic gum solution, the interaction with NMs was conducted at different concentrations (5, 10, 15, and 25 µg/mL for *Candida parapsilosis*). Each experimental condition was evaluated in triplicate. The test tubes were placed in a water bath for 2 h at 35 °C and irradiated by a 23 W incandescent bulb. The tubes were then removed from the water bath, and 100 µL was taken from each tube (fungal suspension exposed to different NMs concentrations), placed on SDA, and dispersed with glass beads via the Copacabana method (by duplicate). The Petri dishes were placed in an incubator for 48 h at 35 °C for subsequent counting. For the *Aspergillus niger* strain, the NMs concentrations were 275, 350, 425, and 500 µg/mL, with a 3 h interaction time at 25 °C. Afterward, 100 µL of the interaction media were dispersed on PDA by duplicate and incubated at room temperature for 36 h. The minimal inhibitory concentration (MIC) and minimal fungicidal concentration (MFC) were parameters to determine the antifungal activity. The MIC was defined as the lowest concentration of NMs that inhibited the fungal growth with respect to the CFU found in the control group. The MFC was defined as the lowest concentration of NMs that completely inhibited fungal growth.

To understand the interaction of the *Candida parapsilosis* and *Aspergillus niger* strains with the synthesized NMs, atomic force microscopy (AFM) and holotomography microscopy (HT microscopy) were used. AFM microscopy was conducted to analyze the NM–fungi interaction. A droplet of 10 µL of the different interaction media was withdrawn at half the interaction time, deposited on a mica substrate functionalized with glycine (0.01 wt.%), and heat fixed. The samples were analyzed on scanasyst mode using a Dimension Edge (Bruker) equipped with a Scan Asyst microscope. The samples were examined in scanasyst mode in air with SCANASYST-AIR tips (silicon tip on Nitride lever; T: 650 nm; f_0_ = 70 kHz; k = 0.4 N/m). Images were recorded using height, adhesion, and peak force channels on square frames of 10 × 10, 5 × 5, and 2.5 × 2.5 µm. HT microscopy was carried out with a sample of 10 µL, placed in a glass substrate, and covered with coverslips. The samples were analyzed with an HT-2L microscope (Tomocube Inc., Daejeon, South Korea).

### 2.5. Cyto-Toxicity Test

**Cell culture.** For the toxicity tests, human bronchial epithelial cells (BEAS-2B) were selected for interaction with the NMs. The cells were obtained from the American Type Culture Collection (CRL-9609). BEAS-2B cells were cultured in Bronchial Epithelial Cell Growth Basal Medium (BEBM™) with all the additives provided by LONZA/Clonetics (Kit Catalog No. CC-3170). Cells were maintained at 37 °C in a humidified atmosphere containing 5% CO_2_. Cells were seeded at 5 × 10^4^ cells/cm^2^ in transwell inserts and allowed to adhere 24 h prior to NMs exposure.

**NMs exposure test.** For the exposure test of BEAS-2B cells to NMs, a stock solution of CuI and TiO_2_-Cu^2+^/CuI with a concentration of 5 mg/mL was made by dispersing the materials in a 1:80 PBS sterile water solution with an ultrasonic bath for 15 min. Afterward, the NM suspensions were nebulized and deposited on the BEAS-2B cells in different concentrations (1.0, 2.5, and 5.0 µg/cm^2^) using an ALI exposure Cloud 6 system from Vitrocell^®^ (Vitrocell^®^, Waldkirch, Germany).

**Cell viability of BEAS-2B.** The cell viability after exposure to NMs of BEAS-2B cells was measured by MTS (methyl tetrazolium salt) metabolism and LDH (lactate dehydrogenase) release assays. After 24 h exposure to CuI and TiO_2_-Cu^2+^/CuI NMs, cell media were taken, and LDH release was evaluated according to the manufacturer protocols (LDH cytotoxicity detection kit Roche, catalog no.: 11644793001). Cells were washed twice with PBS solution; afterward, 1 mL of MTS solution (1:10) in BEBM medium was added to each insert and incubated for 2.5 h. Finally, the medium with the formed formazan was obtained and measured by UV-Vis spectroscopy at 490 nm for MTS in a MODEL spectrophotometer. The results of cell viability tests are represented as the mean± standard error of the mean (SEM) of three experiments in triplicate. The results are compared to a control group and represented as a percentage of the control value.

**Evaluation of the interaction of BEAS-2B and NMs by AFM and HT.** The assessment of the interaction between BEAS-2B cells and CuI or TiO_2_-Cu^2+^/CuI NMs was analyzed by AFM and HT techniques. The morphology of the cells and the interaction of the NMs with the cell membrane were determined by AFM; images were analyzed with the NanoScope Analysis software. The procedure for the AFM sample preparation was as follows: cells were seeded on coverslips before NM exposure. After 24 h of seeding, cells were exposed to 1 µg/cm^2^ of either CuI or TiO_2_-Cu^2+^/CuI NMs. After 24 h, the medium was taken; the cells were fixed using a 10% buffered paraformaldehyde solution and dried with increasing ethanol concentrations. Finally, AFM was conducted in Scanasyst mode.

The internalization and interaction of BEAS-2B cells and CuI or TiO_2_-Cu^2+^/CuI NMs were determined by holotomography microscopy. The sample preparations were as follows: cells were seeded on coverslips for 24 h prior to NM interaction; following this time, cells were exposed to a concentration of 1 µg/cm^2^ of either CuI or TiO_2_-Cu^2+^/CuI NMs. After 3 h of interaction time, the coverslips with cells were washed with PBS solution, and different fluorescent markers were added to identify the mitochondria (MitoTracker™ Green FM, ThermoScientific, Waltham, MA, USA, catalog no.: M7514) and lysosome (LysoTracker™ Red DND 99, ThermoScientific, Waltham, MA, USA, catalog no.: L7528) by fluorescence. Lastly, after adding fluorophores, samples were prepared in PBS solution to be observed in a holotomography microscope (Tomocube, Inc., Daejeon, Republic of Korea).

## 3. Results

### 3.1. Synthesis and Characterization of NMs

TiO_2_-Cu^2+^/CuI nanocomposites were synthesized using a two-step procedure (sol-g-gel followed by controlled precipitation). The suspension of the synthesized nanomaterials (CuI) consists of light cream color suspensions; in the case of the TiO_2_-Cu^2+^/CuI composite, the cream color became darker, and a high yield of the synthesis reaction was observed (>90%). The colloidal suspension shows stability for about a week. 

**Figure 1:**
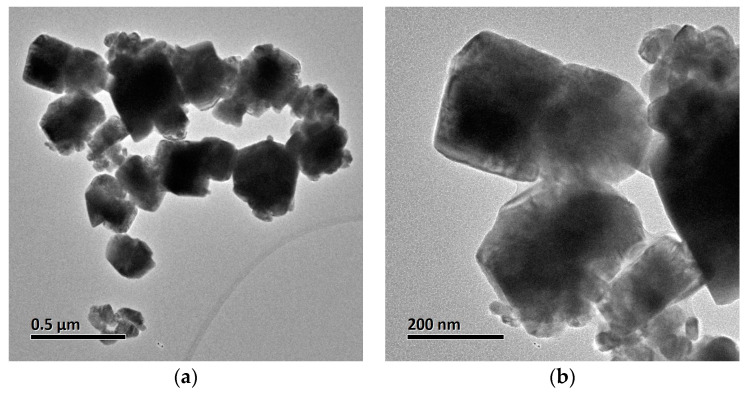

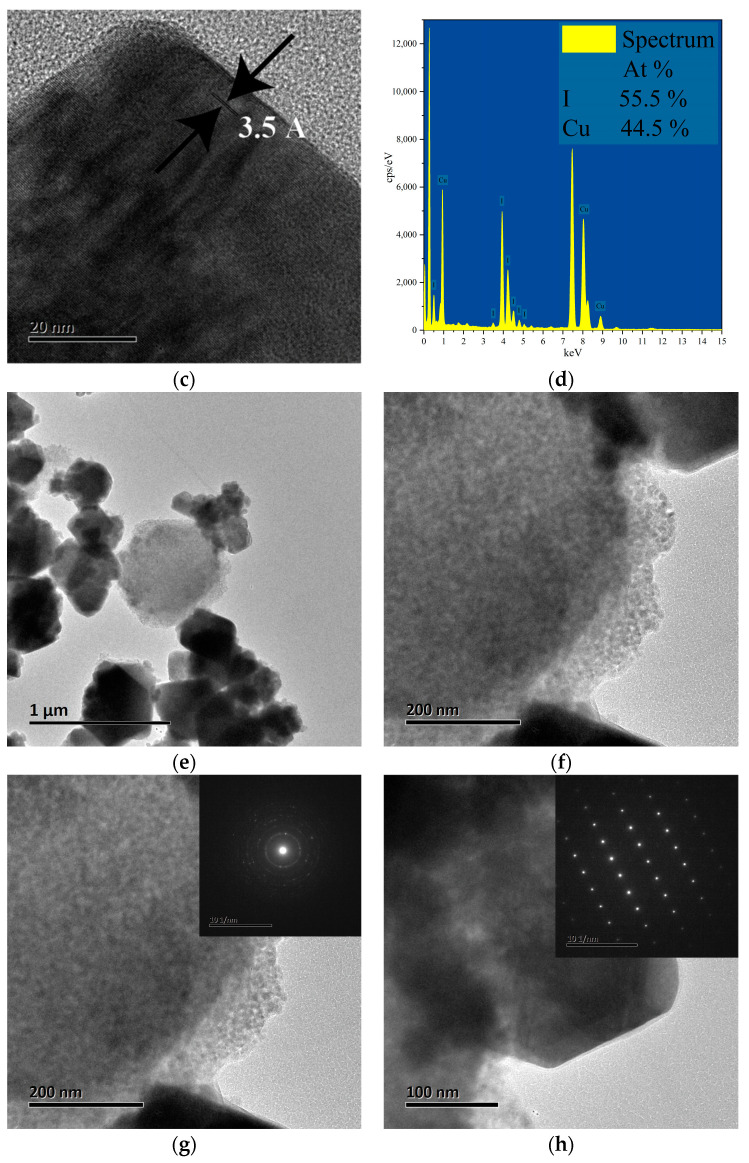
TEM and HRTEM images of the CuI and TiO_2_-Cu^2+^/CuI nanoparticles, (**a**) low-magnification TEM micrograph of CuI powdered material; (**b**) magnification of geometrical shape CuI nanoparticles; (**c**) HRTEM of CuI nanoparticles; (**d**) EDX analysis of CuI; (**e**) low-magnification TEM micrograph of TiO_2_-Cu^2+^/CuI; (**f**) magnification of TiO_2_-Cu^2+^/CuI composite; (**g**) SADP analysis of TiO_2_-Cu^2+^ section of composite; (**h**) SADP analysis of CuI section of composite.

**TEM-EDX.** TEM-EDX analysis was conducted on the dried samples to assess the nanoparticle morphology. The results are shown in Figure 1a–h and Figure S2. As observed from the micrographs, CuI nanoparticles are agglomerated in large, uneven geometrical shapes with sharp edges. The nanoparticles have a mean length of 144 ± 60 nm (*n* = 100 particles); the high standard deviation of the measurements is due to the high size distribution, with a minimum and a maximum size of 21 nm and 315 nm, respectively. The EDX analysis of the samples revealed only the presence of I (55.5%) and Cu (44.5%) in the sample, which is in accordance with the proposed formulae corresponding to the CuI material. Furthermore, in Figure 1c, the HRTEM shows the lattice fringe of the material, and the (1 1 1) plane of the CuI cubic structure was confirmed by measuring the d space, with a value of 3.54 Å [35].

On the other hand, in Figure 1e and Figure S2, the TiO_2_-Cu^2+^/CuI composite shows nanoparticles of equally uneven shape and size (CuI) that are surrounded by smaller sphere-like nanoparticles (TiO_2_-Cu^2+^). EDX analysis (Figure S2) denotes the purity of the composite NMs. The elemental composition of the composite includes Ti, O, Cu, I, and P. The atomic proportions of the elements vary depending on the scanned zone but are in accordance with the composite formulation. The TiO_2_-Cu^2+^ nanoparticles were measured using the ImageJ^®^ software, obtaining an average size of 12 ± 3.6 nm. On the other hand, the CuI particles were agglomerated with no visible edges among them, which results in a mean size of 109 ± 96 nm with a minimum size of 30 nm and a maximum agglomerate size of 390 nm. Furthermore, the HRTEM showed the lattice fringe of CuI and TiO_2_-Cu^2+^; the d spacing measurements were conducted employing ImageJ^®^ software and are shown in Figure S1. The d spacing indicates the presence of TiO_2_-Cu^2+^ and CuI (1 1 1) and (1 0 1) planes. Furthermore, analysis of the selected area diffraction patterns (SADP, Inset Figure 1g,h) corroborates the d spacing values (measured from Digital Micrograph Software) which are shown in Table 1. The CuI d spacing is more significant in the synthesized composite than in the CuI nanoparticles, possibly due to the TiO_2_-Cu^2+^ addition in the composite. There is a clear difference in the atomic arrangement of the two materials. According to the SADP analysis, zones in the material correspond to polycrystalline TiO_2,_ while the CuI appears to be highly crystalline. 

**Figure 2:**
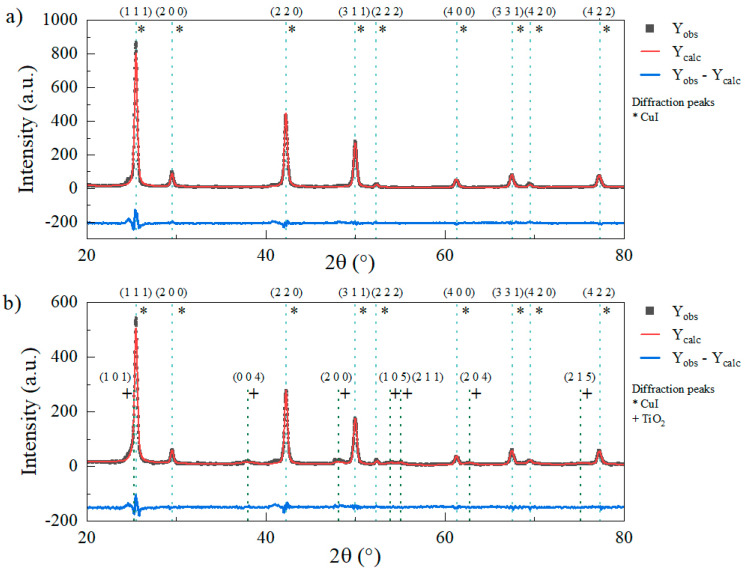
XRD patterns and Rietveld analysis comparison of CuI and TiO_2_-Cu^2+^/CuI powdered materials. (**a**) CuI diffractogram with indicated planes. (**b**) TiO_2_-Cu^2+^/CuI composite diffractogram with CuI and TiO_2_ planes; (**-**) experimental pattern data, (**-**) calculated pattern, (**-**) residual.

**XRD.** XRD analysis was conducted on both samples to assess the crystalline phases and crystallite size and corroborate the SADP results. Rietveld analysis was conducted to assess phase quantification and crystallite size; the comparison of recorded XRD patterns and Rietveld analysis is shown in Figure 2. The peak identification was made by comparison using the powder diffraction file (PDF) database supplied by the ICDD. As can be seen from the CuI diffractogram (Figure 2a), the main diffraction located at 25.5° corroborates the presence of the (1 1 1) plane and cubic crystalline phase (PDF 083-1108). Furthermore, the diffraction peaks located at 29.5°, 42.3°, 50.0°, 52.3°, 61.3°, 67.5°, 69.4°, and 77.2° indicate the presence of cubic structure CuI, according to the PDF file and the literature [15,35,36,37], and are in accordance with the d spacing measurements and planes found by SADP analysis. The crystallite size was evaluated through Williamson–Hall plots [38,39], obtaining an average size of 24.1 nm and a strain value of ε = 0.4 × 10^−3^. On the other hand, in Figure 2b, the diffractogram recorded for the TiO_2_-Cu^2+^/CuI composite shows the same diffraction pattern corresponding to the CuI phase at the same 2θ positions as in Figure 1a. In addition, the main peak corresponding to the TiO_2_-Cu^2+^ anatase phase found at 25.3° of the (1 0 1) plane is covered by the high diffraction peak of the (1 1 1) plane of CuI; nevertheless, the peaks located at 37.9°, 48.1°, 53.9°, 55.1°, 62.7°, and 75.1° are in agreement with the TiO_2_ anatase phase diffraction peaks (PDF 075-2545). Furthermore, the phase quantification of the TiO_2_-Cu^2+^/CuI pattern from the Rietveld analysis (goodness of fit χ^2^ = 0.432) reported a 68.2 wt.% and a 31.8 wt.% of CuI and TiO_2_-Cu^2+^, respectively, which is in accordance with the proposed TiO_2_:CuI ratio of 1:2 when synthesizing the composite. The crystallite size and strain were calculated from Williamson–Hall plots, obtaining an average crystallite size of 27.8 nm and 9.7 nm and a strain value of 1.1 × 10^−3^ and 10.3 × 10^−3^ for the CuI and TiO_2_-Cu^2+^ phases, respectively. The results show an increase in the lattice strain of the CuI component of the composite when compared to the CuI nanoparticles, which could be related to TiO_2_-Cu^2+^ interactions and explain the d spacing increase observed in the SADP analysis [39,40]. Finally, the TiO_2_-Cu^2+^ crystallite size of 9.7 nm is in accordance with the nanoparticles observed in the TEM analysis (Figure 1g).

**Figure 3:**
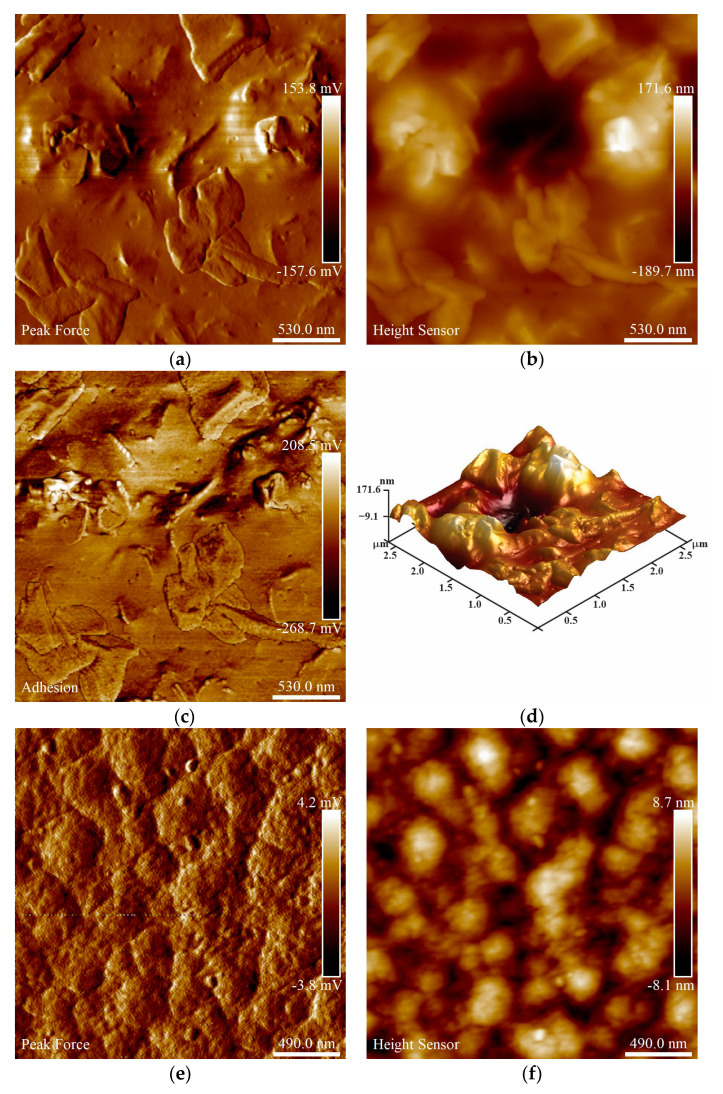

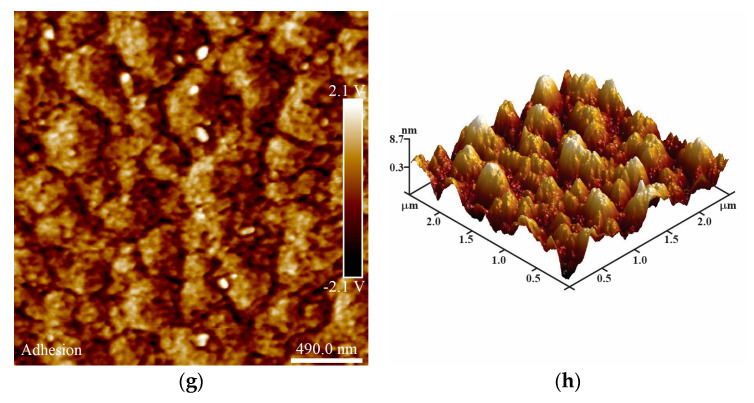
AFM analysis of CuI nanoparticles and TiO_2_-Cu^2+^/CuI composites (**a**–**d**) CuI films; peak force, height, 3D height, adhesion analysis, respectively; (**e**–**h**) TiO_2_-Cu^2+^/CuI films; peak force, height, 3D height, adhesion analysis, respectively.

**AFM.** Surface analysis of the synthesized nanomaterials was conducted by AFM. Thin films of the nanomaterials were prepared by suspending the nanomaterials in Arabic gum and depositing on glass substrates. The AFM images of the CuI nanoparticles and TiO_2_-Cu^2+^/CuI composites are shown in Figure 3. According to the images recorded, the CuI nanoparticles (Figure 3a–d) show large agglomerates of nanoparticles with different shapes and sizes with an average thickness of 170 nm. Furthermore, the surface morphology reveals the same geometrical shape and clear edges of the nanoparticles observed by TEM analysis. On the other hand, Figure 3e–h showed that the TiO_2_-Cu^2+^/CuI composites are evenly distributed throughout the surface. Moreover, according to the height sensor image (Figure 3f,h), the composite film is approximately ≈ 9 nm thick, and it is important to notice that in the composite films, the geometrical shape of the CuI was not observed.

**DLS.** The dynamic light scattering technique was used to determine the polydispersity index, hydrodynamic diameter, and zeta potential, shown in Table 2. The CuI NMs showed a hydrodynamic diameter of 85.41 ± 2.37 nm in water, with a polydispersity index of 0.434 and a zeta potential of −51.9 ± 10.71 mV. On the other hand, the corresponding values of the TiO_2_-Cu^2+^/CuI NMs were 234.07 ± 3.33 nm, 0.455, and −35.87 ± 1.31, respectively. The zeta potential values corroborate the stability of the NMs in the suspension. However, the composite shows a larger hydrodynamic diameter, indicating the agglomeration of the particles in an aqueous solution. Furthermore, the large polydispersity index agrees with size variation and agglomeration observed in the TEM and AFM surface analysis.

### 3.2. Antifungal Activity of CuI-Based Nanostructured Materials

#### 3.2.1. Candida Parapsilosis Interaction Tests

Several parameters, such as exposure time and NM concentration, were evaluated to optimize the interaction test of CuI-based materials and pathogenic fungi. Following previous research with similar materials, a first attempt was made with different concentrations of NMs (5, 10, 15, 25, 50, and 75 µg/mL) [17]. According to these preliminary results, the antifungal activity of CuI and TiO_2_-Cu^2+^/CuI composites is inhibited due to the aggregation of NMs as concentrations increase, thus decreasing the interaction of NMs and fungi and limiting the antifungal activity (see Figure S3a,b).

**Figure 4:**
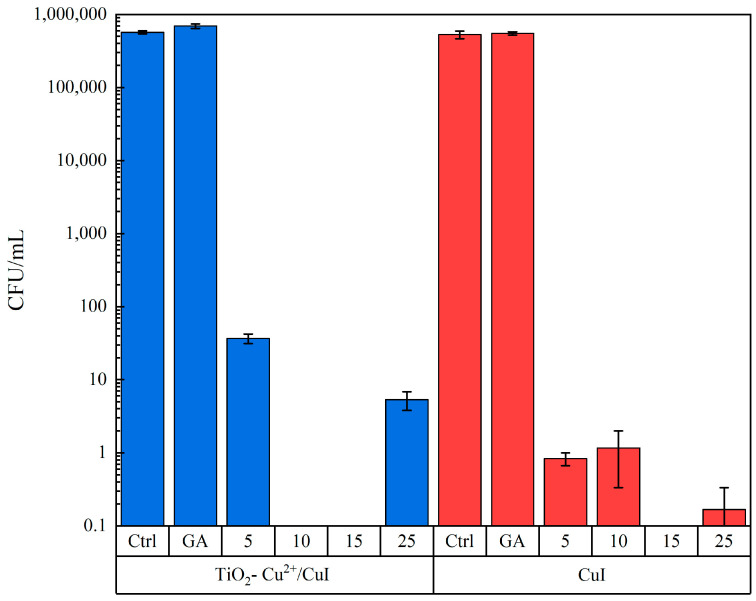
Antifungal activity of TiO_2_-Cu^2+^/CuI and CuI to inhibit *Candida parapsilosis* growth: control group (Ctrl); Arabic gum control (GA); 5 µg/mL NMs (5); 10 µg/mL NMs (10); 15 µg/mL NMs (15); 25 µg/mL (25).

To avoid possible reproducibility errors due to NM aggregation, interactions with fungi were evaluated at the following concentrations: 5, 10, 15, and 25 µg/mL and with short exposure times (2 h), in addition, each test was compared with a control group and a control group adding Arabic gum to discard any possible effect from the dispersant. With these experimental conditions, the two tested NMs have similar antifungal activity (the MIC is the same at concentrations <5 µg/mL). Moreover, as observed in Figure 4, the inhibition in both cases is over 99.99% at 2 h of interaction with a minimal concentration of the materials; it is important to notice that the y-axis of the graphic is illustrated in the log scale to observe the difference in the CFU count better (see Figure S3c–n).

Furthermore, our results indicate an MFC of 10 µg/mL and 15 µg/mL for TiO_2_-Cu^2+^/CuI composites and CuI NMs, respectively, which means that complete inhibition is achieved at low NM concentrations and that the TiO_2_-Cu^2+^/CuI composite enhances its antifungal properties against certain microbial strains. Several investigations report the antibacterial properties of CuI NMs and their efficiency depending on the bacterial strain. For instance, Pramanik et al. [24] studied the antibacterial properties of CuI and reported a MIC value of 0.1 mg/mL for *Escherichia coli*, an MBC (minimal bactericidal concentration) of 0.11 mg/mL, and a value of 0.1 and 0.15 mg/mL for MIC and MBC against the *Staphylococcus aureus* strain. Additionally, Awed et al. described the antimicrobial activity of CuI against several bacterial strains using disc diffusion tests. Their results demonstrate the antibacterial activity of CuI at low doses; the susceptibility to the NMs varies with the biological entities. Previous studies from our research group investigated the antifungal activity of CuI against *Candida albicans* and *Sporothix schenckii* [17]. In this study, we demonstrate the antifungal activity of CuI against *Candida parapsilosis* at low doses and short exposure times. However, we aim to develop robust NMs that exert antifungal activity through diverse mechanisms; TiO_2_-Cu^2+^/CuI. Our results demonstrate the enhanced antifungal activity of the TiO_2_-Cu^2+^/CuI composite; we can observe that the incorporation of TiO_2_ enhanced the CuI activity, thus obtaining a lower MFC of 10 µg/mL, attributed to the TiO_2_-Cu^2+^ antimicrobial activity and capacity to form reactive oxygen species (ROS) [11,41].

#### 3.2.2. Aspergillus Niger Interaction Tests

**Figure 5:**
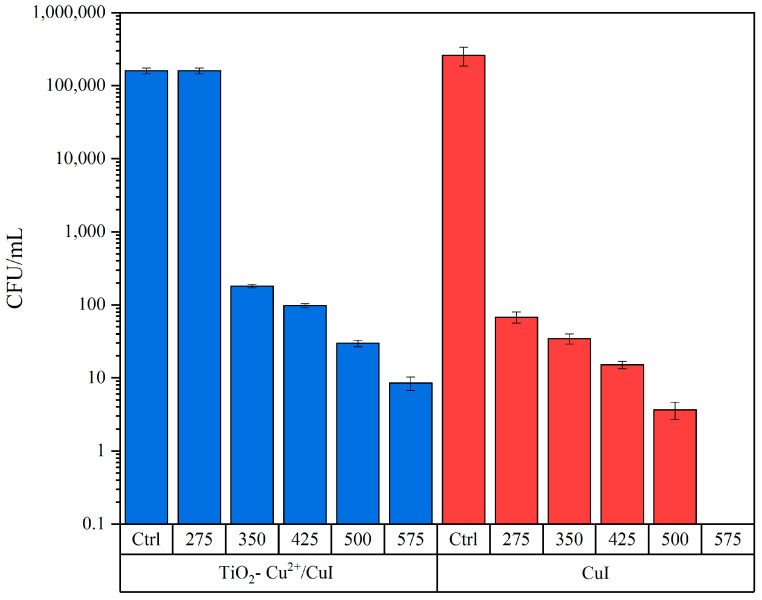
Antifungal activity of TiO_2_-Cu^2+^/CuI and CuI to inhibit *Aspergillus niger* growth: control group (Ctrl); 275 µg/mL NMs (275); 350 µg/mL NMs (350); 425 µg/mL NMs (425); 500 µg/mL (500); 575 µg/mL (575).

Following the antifungal test of the NMs, the *Aspergillus niger* strain was used as a model to show the antifungal properties of CuI and TiO_2_-Cu^2+^/CuI. Preliminary tests using the same concentrations that were used with the *Candida parapsilosis* strain were conducted with different interaction times (3 and 6 h). According to the preliminary test, there is no inhibition of *Aspergillus niger* growth at either 3 or 6 h of interaction time. Therefore, the concentrations used to assess the antifungal activity were increased to 275, 350, 425, 500, and 575 µg/mL with an interaction time of 3 h; the results can be observed in Figure 5 and Figure S4. The interaction tests were compared to a control group of the fungi with the same treatment as the interaction; the Arabic gum control group was not added since it was proven there is no effect due to the dispersant.

According to the results in Figure 5, the CuI has a MIC of 275 µg/mL, while the MIC for the TiO_2_-Cu^2+^/CuI composite is 350 µg/mL. On the other hand, the MFC for CuI was 575 µg/mL; furthermore, the composites do not totally inhibit the growth of *A. niger*. This result can be due to the aggregation and precipitation of composite NMs at higher concentrations in the exposure medium. However, the materials under study exert higher antifungal activity by comparing to previously synthesized CuI composites; for instance, Ghanbari and Salavati-Niasari [42] studied the CuI/C_3_N_4_ composite and its antifungal activity against different microorganisms, including *Aspergillus niger* and *Candida albicans*. According to their results, the composite did not display any antimicrobial activity against these two microorganisms.

Likewise, different types of NMs have been studied and display antifungal activity against *Aspergillus niger*. Sawangphruk et al. [43] utilized reduced graphene oxide nanosheets to inhibit *Aspergillus niger* fungi growth with an MFC of 500 µg/mL. Sayed et al. [44] reported the antifungal activity of several nanoferrite structures and stated MIC values > 100 µg/mL for silver ferrite.

#### 3.2.3. AFM and HT Evaluation of the Interaction of Fungi and NMs

**Figure 6:**
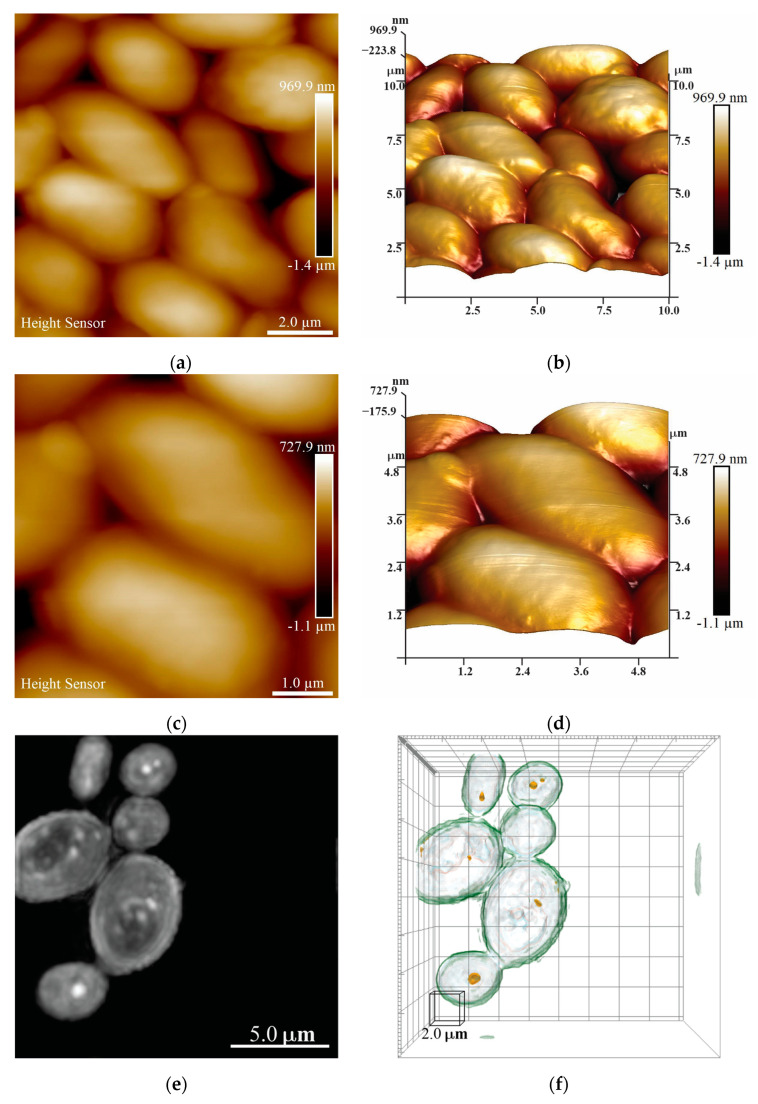

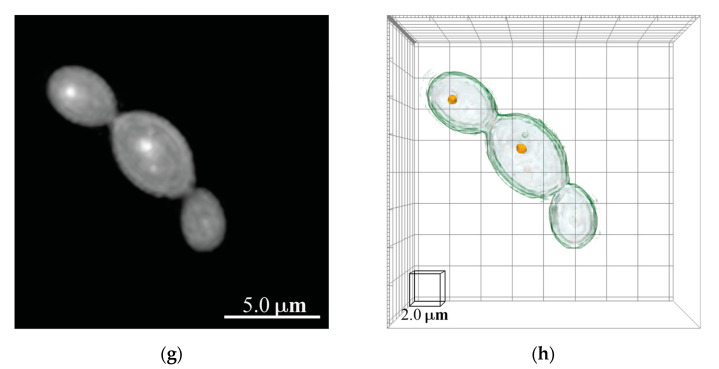
AFM and HT microscopy analysis of *Candida parapsilosis* control group: (**a**) AFM height sensor image; (**b**) 3D view of the height sensor; (**c**) height sensor magnification image; (**d**) 3D view of the magnification zone; (**e**,**g**) RI tomogram of *Candida parapsilosis* control group; (**f**,**h**) 3D reconstruction image.

The interaction of fungi strains and NMs was evaluated by AFM and HT microscopy. The control group of *Candida parapsilosis* can be seen in Figure 6. As observed from AFM micrographs, cell colonies can be found and are in accordance with morphology and size corresponding to the *Candida* yeast strain, that is, an oval shape and 3–4 µm size [45,46]. In addition, in HT microscopy, a relatively novel microscopy technique that enables 3D reconstruction based on refraction index, the colonies of *Candida parapsilosis* can be observed at the RI tomogram and are in accordance with the AFM analysis in the shape and size of the yeast strain.

**Figure 7:**
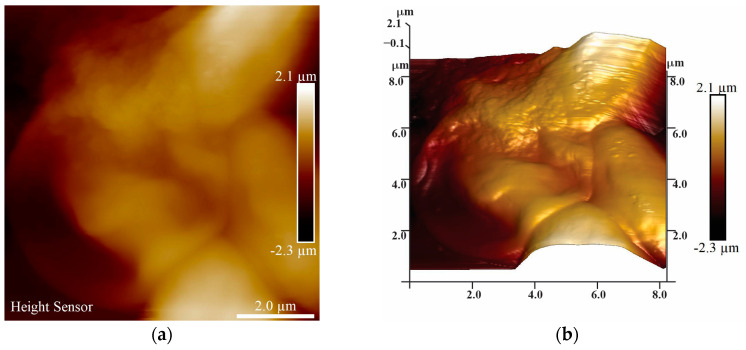

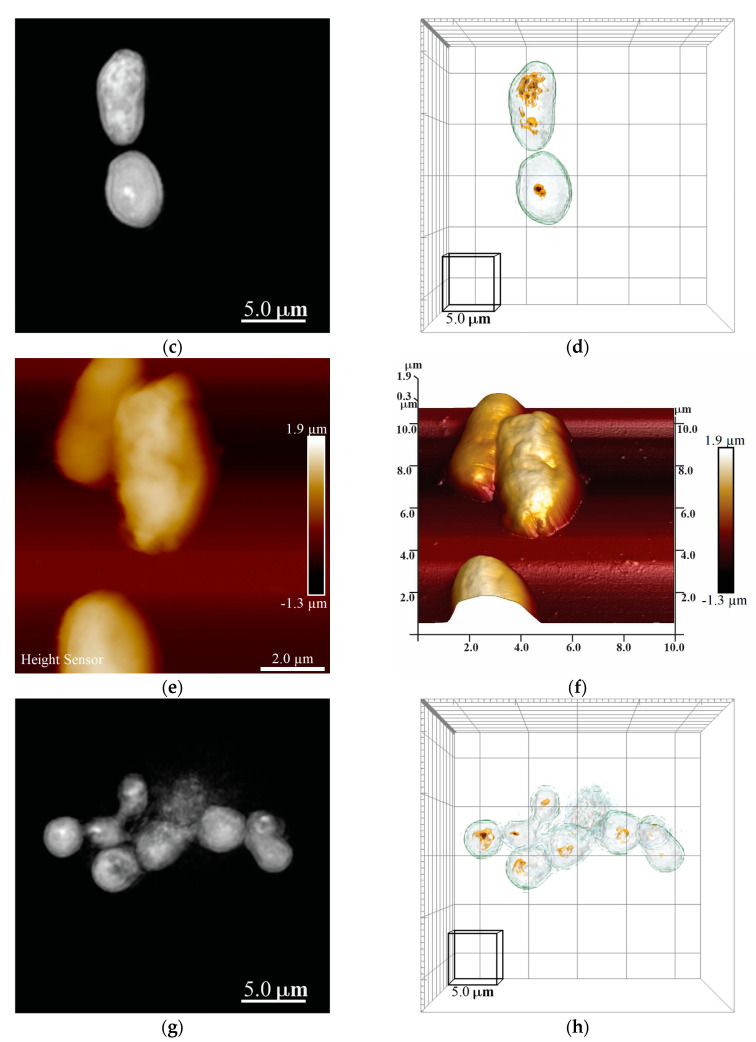
AFM and HT microscopy analysis of *Candida parapsilosis*–NM interaction: (**a**,**b**) AFM height sensor and 3D reconstruction of CuI interaction; (**c**,**d**) RI tomogram and 3D reconstruction of *Candida parapsilosis–*CuI interaction; (**e**,**f**) AFM height sensor and 3D reconstruction of TiO_2_-Cu^2+^/CuI interaction; (**g**,**h**) RI tomogram and 3D reconstruction of *Candida parapsilosis–*TiO_2_-Cu^2+^/CuI interaction.

The AFM and HT microscopy analysis conducted after the yeast–NM interaction is shown in Figure 7. It is crucial to notice that these samples were taken at half-time of the interaction test to ensure that there were still cells in the interaction medium and that the interaction between the cells and NMs was detectable. First, the AFM analysis shows that the *Candida parapsilosis* cells are no longer in large clusters. Moreover, the cells are deformed, and some visible deposits of NMs can be seen on the surface of the cells. The same effect was observed for both TiO_2_-Cu^2+^/CuI and CuI NMs (see Figure 7a,b,e,f). It is essential to notice that the AFM and HT microscopy samples compared in Figure 7 were taken from the same batch experiment. Continuing with the analysis, in the RI tomogram of the interaction, the cells are deformed. Employing the 3D reconstruction, it is possible to observe the higher diffraction index as a red color in the 3D view of the cells, which is attributed to the NMs interacting with the yeast cells. We can observe that the NMs are inside the cells and even promote membrane rupture (see Figure 7c,d,g,h). Further comparison of the *Candida parapsilosis* control group and interaction with NMs can be observed in Figure S5, where the differences between the control group are visible, both in the shape and size of the yeast after the interaction and the membrane rupture of the cell. Therefore, this proves that NMs can penetrate the fungal membrane and cause damage by ROS production, as previously reported by Pramanik et al. [24].

### 3.3. Toxicity Tests of CuI and TiO_2_-Cu^2+^/CuI Composite

**Figure 8:**
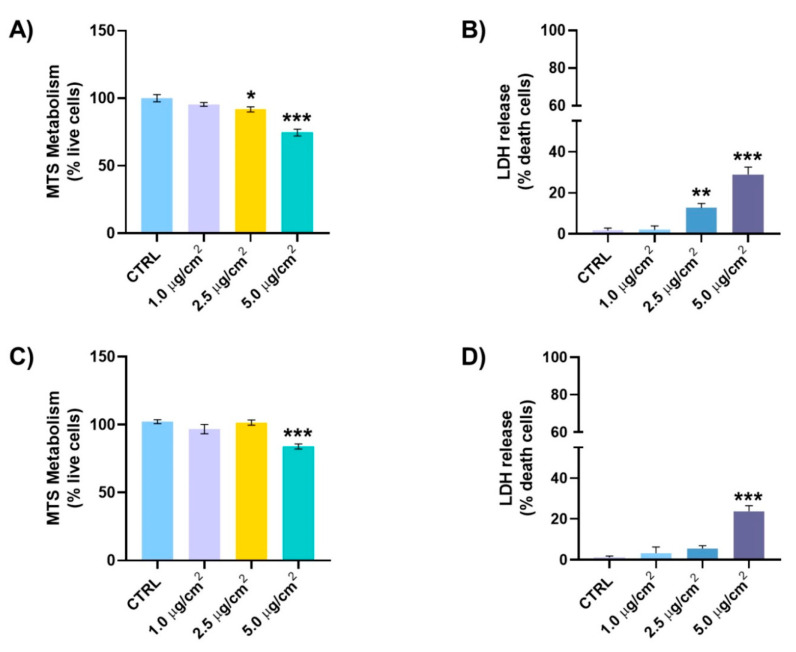
BEAS-2B cells viability determination after CuI or TiO_2_-Cu^2+^/CuI exposure. After 24 h of exposure, the MTS metabolism (**A** or **C**) and LDH release (**B** or **D**) were determined in the BEAS-2B cells. The results of the CuI NPs (**A**,**B**) and the TiO_2_-Cu^2+^/CuI (**C**,**D**) are shown. The experiments were performed in triplicate. Each bar represents the mean ± SEM; * *p* < 0.05, ** *p* < 0.01, *** *p* < 0.001 versus untreated cells. One-way ANOVA, post hoc Bonferroni.

The cell viability was assessed to identify possible cytotoxic damage generated by CuI or TiO_2_-Cu^2+^/CuI nanoparticles after 24 h of interaction with BEAS-2B cells using a bronchial biological model and a realistic inhalation approach by the ALI system. Figure 8A (MTS assay) and Figure 8B (LDH assay) show the viability assay after CuI exposure. According to the results, the cell viability of BEAS-2B decreased to 75 and 91% live cells and 12 and 27% death cells with a concentration of 2.5 and 5.0 µg/cm^2^, respectively; the decrease in the MTS metabolism and the increase in the LDH liberation shows concentration-dependent cytotoxicity in both cases. On the other hand, the viability of BEAS-2B cells after interaction with TiO_2_-Cu^2+^/CuI shows a cytotoxic effect at higher concentrations (5.0 µg/cm^2^). MTS assay (Figure 8C) shows a significant decrease (22% death cells) at 5.0 µg/cm^2^; in addition, a significant release of LDH (83% live cells) was observed at the same concentration (Figure 8D). These results suggest a higher cytotoxic effect produced by CuI nanoparticles compared to the TiO_2_-Cu^2+^/CuI nanoparticles.

#### HT Microscopy Evaluation of the Interaction of BEAS-2B and NMs

**Figure 9:**
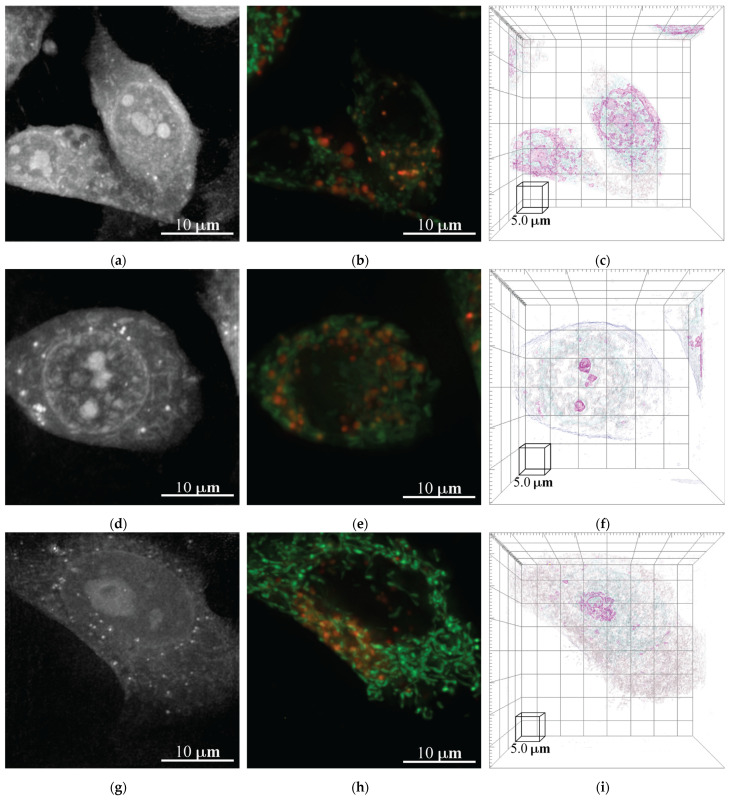
HT microscopy evaluation of BEAS-2B–CuI and TiO_2_-Cu^2+^/CuI NM interaction: RI tomogram, fluorescence marker, and 3D reconstruction of (**a**–**c**) BEAS-2B control group; (**d**–**f**) BEAS-2B–CuI interaction; (**g**–**i**) BEAS-2B–TiO_2_-Cu^2+^/CuI interaction.

Following the cytotoxic damage analysis, HT microscopy was conducted to evaluate the BEAS-2B–NM interaction. The damage was assessed at a higher concentration of 5.0 µg/cm^2^. In Figure 9a–c and Figure S6, we can observe the control group of the BEAS-2B cell: an RI tomogram, a 3D reconstruction view, the mitochondria, and lysosome fluorescence markers in green and red, respectively. The control group, which is in accordance with BEAS-2B cell morphology, can be observed in Figure 9a–c. Furthermore, the distribution of the lysosomes and mitochondria can be observed in the merged image. When compared to the images of the treated cells, we can observe an evident morphology change and a modification in the mitochondria and lysosome distribution inside the BEAS-2B cell, which is indicative of the cytotoxic damage caused by the interaction with NMs and is in accordance with the MTS and LDH assays (Figure 9d–i).

After exposure to CuI NMs, HT analysis shows changes in the cell morphology and organelle distribution; however, there is no presence of the CuI NMs. This observation supports the enhanced toxicity of CuI, due to dissolution and ion lixiviation [47]. It is important to remark that the NMs under study exert antifungal activity at short exposure times (2 or 3 h), thus decreasing the probability of CuI NM dissolution. Moreover, using HT analysis, we demonstrated the uptake of NMs (TiO_2_-Cu^2+^-CuI) by fungal and BEAS-2B cells. After the internalization of the NMs in the cells, their fate depends on the microenvironment of the cell (pH) and the cell’s biological response [48].

**Figure 10:**
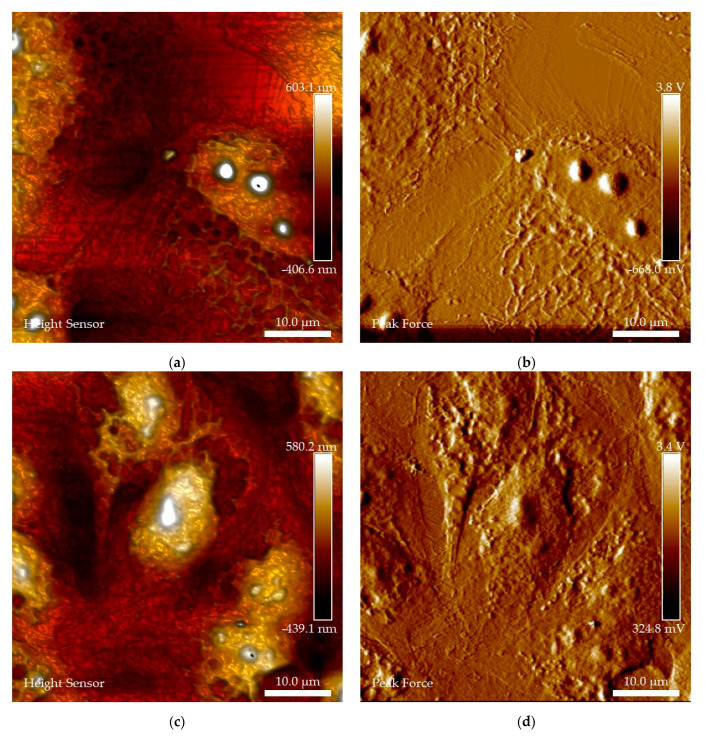

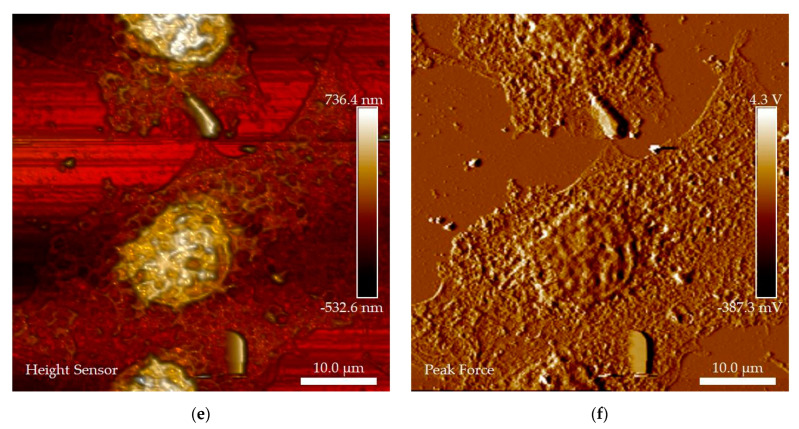
AFM microscopy evaluation of BEAS-2B–CuI and TiO_2_-Cu^2+^/CuI NMs interaction, height sensor images: (**a**,**b**) BEAS-2B control group; (**c**,**d**) BEAS-2B–CuI interaction; (**e**,**f**) BEAS-2B–TiO_2_-Cu^2+^/CuI interaction.

Furthermore, AFM microscopy was conducted to analyze the surface of the BEAS-2B cells with a height sensor, and peak force images were recorded. The height sensor and peak force images in Figure 10a,b and Figure S7 show the control group and are in accordance with the size and morphology of the BEAS-2B cell; the bright surface dots correspond to the nucleoli of the cells and correspond with HT microscopy. After interaction with CuI (Figure 10c,d and Figure S8), a change in morphology can be observed, along with NM deposits on the membrane surface. In addition, in Figure 10c and Figure 8A, a rounded damaged cell can be observed; furthermore, the nucleoli distribution is changed and even absent in some cells. Figure 10e,f and Figure S9 show the BEAS-2B cells after the TiO_2_-Cu^2+^/CuI interaction. It is possible to observe NMs deposits on the surface of the membrane, as well as clear changes in morphology and nucleoli, which were also observed by HT microscopy and are clearly indicative of the damage caused by the NMs.

## 4. Discussion

This research reports the antifungal activity and cytotoxicity of CuI and the TiO_2_-Cu^2+^/CuI composite. The synthesis of the composite NMs undergoes the co-precipitation method, rendering stable colloidal suspensions, which allows their use as prepared without further processing. Previous reports rely on the drying and suspending of NMs to evaluate their antibacterial activity [17,49]. Furthermore, earlier investigations demonstrate the suitability of TiO_2_/CuI NMs for biomedical and environmental applications, including as antibacterial agents, for dye removal, and as catalysts for methanol oxidation or amidation [30,32,35,36]. This study focuses on the antifungal and biocompatibility of the NMs to later apply them for air purification; however, some other applications can be explored. 

In this sense, this research investigates the antifungal activity application of NMs against a yeast strain and a fungi strain. The composition of the composite under study (TiO_2_, Cu^+^, Cu^2+^, I^−^) offers numerous possibilities to inhibit fungal growth. Previous studies remark that the antibacterial mechanism of CuI is due to ROS-induced DNA damage and membrane rupture [16,24]. For example, Archana et al. [15,16] observed membrane damage and morphology changes in *Escherichia coli* and *Staphylococcus faecalis* after interaction with CuI NMs. Additionally, Pramanik et al. [24] reported membrane disruption on *Bacillus subtilis* and *DH5α* strains after interaction with CuI. 

In this study, we combine traditional microbiology and toxicology assays with advanced microscopy techniques to investigate the mechanism of the bioactivity of the NMs under study. AFM images support membrane damage of the NMs in fungal cells (Figure 7a,b,e,f); furthermore, with the aid of HT microscopy (see Figure 7c,d,g,h), we demonstrate the entry of NMs in the fungal cells. We postulate enhanced antimicrobial activity of the TiO_2_-Cu^2+^/CuI composite since, upon irradiation, electron-hole pairs are generated and react with oxygen and water adsorbed in the surface, resulting in the formation of ^•^O^2−^ and ^•^OH radicals that can directly decompose cell membranes, thus enhancing the fungal inhibition process [8,11,50].

Moreover, the synthesized NMs exhibit concentration-dependent and strain-specific antifungal activity, with the *Aspergillus niger* fungus being more resistant than the *Candida parapsilosis* strain. The difference in susceptibility to NMs’ bioactivity is due to the complex structure of *A. niger* compared to *C. parapsilosis* (multicellular filamentous vs. unicellular fungi). There are not many investigations regarding the antifungal activity of NMs at low doses and short exposure times. For instance, preceding research from our group shows the excellent antifungal activity of CuI against *Candida albicans* and *Sporothrix schenckii*. We also reported that CuI inhibits the growth of *Fusarium oxysporum* but at higher doses and longer exposure times [17]. Some other studies remark on the antifungal activity of silver-based NMs [20]; however, there are concerns regarding the biocompatibility of this material at high doses. Moreover, silver-based materials are not optimal (cost-related) for large-scale applications (air purification).

Following the approach of using traditional microbiology and advanced microscopy techniques to investigate the antifungal activity of the NMs; cytotoxicity evaluation encompasses traditional toxicology and advanced microscopy techniques. As previously stated, an accidental release of NMs might cause adverse health effects in living organisms; thus, we evaluate the possible health effects of the NMs when in contact with human BEAS-2B cells. Our results show the biocompatibility of NMs at low concentrations; however, at high concentrations (>5.0 µg/cm^2^), cell damage was observed by MTS and LDH tests. These results are in agreement with the observations using HT and AFM microscopy, which show membrane damage and changes in the nucleoli, mitochondria, and lysosome distribution after the NM interaction. For example, using AFM, it is possible to visualize the deposition of NMs (composites, Figure 10e,f) on the surface of the cell membrane as well as morphology damage and changes in the distribution of the nucleoli of the cells. Furthermore, using HT, we can visualize changes in organelle abundance and distribution. 

Our results indicate the lower biocompatibility of the CuI NMs (a higher LDH release compared to TiO_2_-Cu^2+^/CuI). This result supports our previous statement regarding TiO_2_-Cu^2+^ mediating the CuI interaction with the cell membrane. AFM images show the presence of the composites in the cytoplasm and their accumulation around the nuclear membrane. It also illustrated damage to the nuclear membrane and changes in the cell morphology. These changes are associated with oxidative damage mainly produced by Cu^2+^ ion release since copper is a redox-active metal. Furthermore, there are no previous reports on the cytotoxicity effects on BEAS-2B cells using a liquid–air interface (ALI) model for the NMs under study. Murugadoss et al. [51] reported the cytotoxic effect of TiO_2_ agglomerates of 117 nm on human epithelial cells using an ALI system and found that low doses (1.62 µg/cm^2^) do not exhibit cytotoxic effects. Nevertheless, they found concentration-dependent cytotoxicity at high doses (up to 38.5 µg/cm^2^). On the other hand, Diabaté et al. [52] exposed human alveolar epithelial cells (A549) to a low dose of TiO_2_ nanoparticles (47 nm). They report moderate LDH release in A549 cells exposed to doses of ~0.2 µg/cm^2^ of TiO_2_. The damage significantly increased at doses of ~1.0 µg/cm^2^. In addition, Hufnagel et al. [53] compared the cytotoxicity of copper- and titanium-based NMs on A549 human cells using an ALI interaction model. According to the research, TiO_2_ does not exert toxicity; however, CuO exhibits a concentration-dependent cytotoxic effect. Furthermore, there are reports on the increase in ROS species due to NM interaction, such as TiO_2_ and CeO_2,_ on BEAS-2B cells [54,55]. In comparison, in this work, the CuI exhibits a slightly higher cytotoxic effect than the TiO_2_-Cu^2+^/CuI composite, detecting cytotoxic effects at concentrations of 2.5 and 5.0 µg/cm^2^, respectively. This effect could be due to CuI dissolution in the exposure media. We must remark that we aim to apply the NMs in the solid state, avoiding the release of ions due to dissolution. 

Lastly, the cytotoxic effects of CuI and TiO_2_-Cu^2+^NMs were assessed by HT microscopy. It is relevant to discuss that HT microscopy reveals the internalization of NMs in fungal cells. For cytotoxicity evaluation, it is not possible to visualize NMs inside cells; however, changes in the morphology and organelle distribution support the damage exerted by the NMs in the BEAS-2B cells. Our results suggest that at low exposure times (1h for antifungal activity), it is possible to visualize the internalization of the NMs. However, for the cytotoxicity study, the cells were exposed to NMs at high exposure times. The HT microscopy analysis reveals changes in the morphology of the cells and the clearance of the NMs to avoid damage (changes in organelle distribution).

## 5. Conclusions

In this work, we presented a systematic study of the synthesis, characterization, bioactivity, and biocompatibility of CuI and TiO_2_-Cu^2+^/CuI NMs. The NMs were synthesized as colloids using a reproducible co-precipitation method (CuI) and a two-step procedure (sol-gel and co-precipitation; TiO_2_-Cu^2+^/CuI). The NMs’ composition (Cu^+^, Cu^2+^, TiO_2_, I^−^) favors the antifungal activity of the NMs at low doses. The NMs have excellent antifungal activity against *Candida parapsilosis* at low doses (<5 and 10 µg/mL) and short exposure times. The antifungal activity of the NMs is suitable to inhibit the growth of filamentous fungi at moderate doses (between 350 and 575 µg/mL), and they are, to our knowledge, one of the few NMs that could inhibit the *Aspergillus niger* strain. In this work, we demonstrated the advantages of using traditional toxicity assays and advanced microscopy techniques to assess the bioactivity and biocompatibility of the NMs. HT microscopy is a suitable technique to investigate the entry and activity of NMs in living cells (fungal or BEAS-2B). Furthermore, AFM provides high-resolution images revealing morphological changes in cells due to NM interaction. The NMs are biocompatible at low doses and are stable and excellent antifungal agents, properties that suit them as potential candidates as precursors to indoor air purification technologies.

## Figures and Tables

**Table 1:** SADP d spacing measurements of TiO_2_-Cu^2+^/CuI.

TiO_2_-Cu^2+b^		CuI	
d Spacing (Å)	Plane	d Spacing (Å)	Plane
3.68	(1 0 1)	3.70	(1 1 1)
2.47	(0 0 4)	2.26	(2 2 0)
1.98	(2 0 0)	1.93	(3 1 1)
1.78	(1 0 5)	1.44	(3 3 1)
1.53	(2 1 1)	1.23	(4 2 2)

**Table 2:** CuI and TiO_2_-Cu^2+^/CuI NMs suspension characterization.

NPs	Hydrodynamic Diameter (nm)	Polydispersity Index	Zeta Potential (mV)
CuI	85.41 ± 2.37	0.434	−51.9 ± 10.71
TiO_2_-Cu^2+^/CuI	234. 07 ± 3.33	0.455	−35.87 ± 1.31

Values were obtained from 1 experiment in triplicate.

## Data Availability

Additional data is available per request.

## Supplementary Materials

The following supporting information can be downloaded at: https://www.mdpi.com/article/10.3390/nano13131900/s1, Figure S1: HRTEM images of TiO_2_-Cu^2+^/CuI composite; (a) HRTEM and d spacing measurement of TiO_2_-Cu^2+^ magnification; (b) HRTEM and d spacing measurement of CuI magnification; Figure S2: TEM (A, B, and C) and EDX (A1, B1, and C1) analysis of composite (TiO_2_-Cu^2+^/CuI) NMs. TEM micrographs show the presence of crystalline CuI surrounded by TiO_2_-Cu^2+^. EDX (A1, B1, C1) results show the purity and composition of the composite NMs; Figure S3: *C. parapsilosis* culture on agar after NM interaction; (a) 1 h preliminary test with TiO_2_-Cu^2+^/CuI composite; (b) 1 h preliminary test with CuI; (c–h) *C. parapsilosis* vs. TiO_2_-Cu^2+^/CuI composite 2 h interaction; (c) control group diluted for counting; (d) GA group diluted for counting; (e) 5 µg/mL TiO_2_-Cu^2+^/CuI; (f) 10 µg/mL TiO_2_-Cu^2+^/CuI; (g) 15 µg/mL TiO_2_-Cu^2+^/CuI; (g) 25 µg/mL TiO_2_-Cu^2+^/CuI; (i–n) *C. parapsilosis* vs. CuI NMs 2 h interaction; (i) control group diluted for counting; (j) GA group diluted for counting; (k) 5 µg/mL CuI; (l) 10 µg/mL CuI; (m) 15 µg/mL CuI; (n) 25 µg/mL CuI; Figure S4: *A. niger* culture on agar after NM interaction; (a–f) interaction test series against TiO_2_-Cu^2+^/CuI composite; (a) *A. niger* control group diluted for counting; (b) 275 µg/mL TiO_2_-Cu^2+^/CuI; (c) 350 µg/mL TiO_2_-Cu^2+^/CuI; (d) 425 µg/mL TiO_2_-Cu^2+^/CuI; (e) 500 µg/mL TiO_2_-Cu^2+^/CuI; (f) 575 µg/mL TiO_2_-Cu^2+^/CuI; (g–l) Interaction test series against CuI; (g) *A. niger* control group diluted for counting; (h) 275 µg/mL CuI; (i) 350 µg/mL CuI; (j) 425 µg/mL CuI; (k) 500 µg/mL CuI; (l) 575 µg/mL CuI; Figure S5: HT microscopy analysis of *Candida parapsilosis*–NM interaction: (a, b) RI tomogram and 3D reconstruction of *Candida parapsilosis* control group; (c,d) RI tomogram and 3D reconstruction of *Candida parapsilosis*–CuI interaction; (e,f) RI tomogram and 3D reconstruction of *Candida parapsilosis*–TiO_2_-Cu^2+^/CuI interaction; Figure S6: HT microscopy evaluation of BEAS-2B–CuI and TiO_2_-Cu^2+^/CuI NMs interaction: RI tomogram, fluorescence marker, and 3D reconstruction of (a–c) BEAS-2B control group; (d–f) BEAS-2B–CuI interaction; (g–h) BEAS-2B–TiO_2_-Cu^2+^/CuI interaction; Figure S7: AFM microscopy evaluation of BEAS-2B control group: (a,c,e) BEAS-2B height sensor; (b,d,f) BEAS-2B peak force; Figure S8: AFM microscopy evaluation of BEAS-2B–CuI interaction test: (a,c,e) BEAS-2B height sensor; (b,d,f) BEAS-2B peak force; Figure S9: AFM microscopy evaluation of BEAS-2B–TiO_2_-Cu^2+^/CuI interaction test: (a,c,e) BEAS-2B height sensor; (b,d,f) BEAS-2B peak force.
