# Supplementary material for: Synthesis of TiO2-Cu2+/CuI Nanocomposites and Evaluation of Antifungal and Cytotoxic Activity
Source: Nanomaterials (Basel). 2023 Jun 21;13(13):1900. doi: 10.3390/nano13131900 (PMC10343807; doi:10.3390/nano13131900)
Supplement: Supplementary file 1 [file nanomaterials-13-01900-s001.zip › nanomaterials-2438397-supplementary.pdf]

Supplementary Materials

Synthesis of TiO_2-Cu^{2+}/CuI Nanocomposites and Evaluation of Antifungal and Cytotoxic Activity

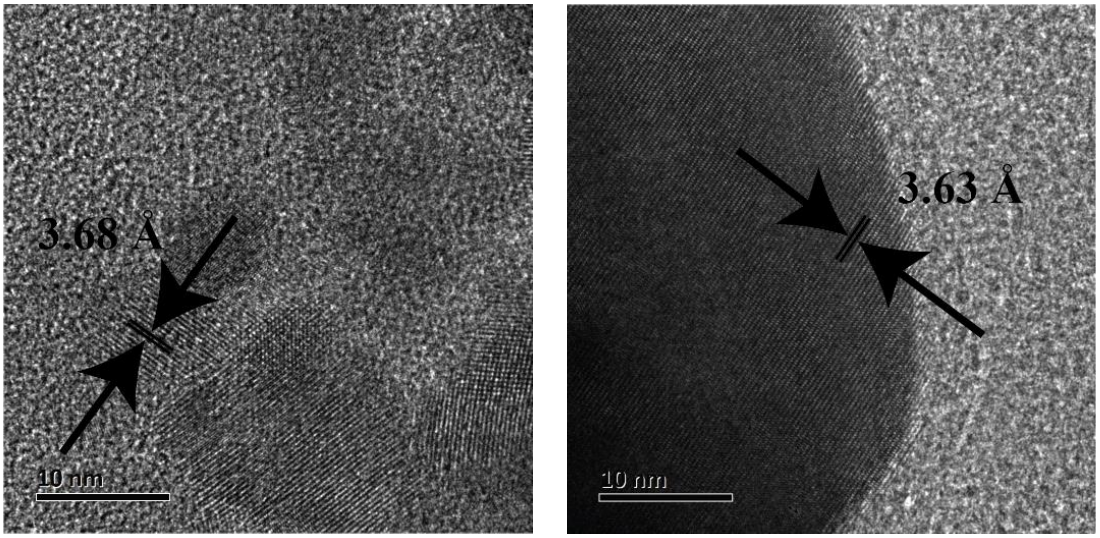

(**a**) (**b**)

Figure S1. HRTEM images of TiO_2-Cu^{2+}/CuI composite; (**a**) HRTEM and d spacing measurement of TiO_2-Cu^{2+} magnification; (**b**) HRTEM and d spacing measurement of CuI magnification.

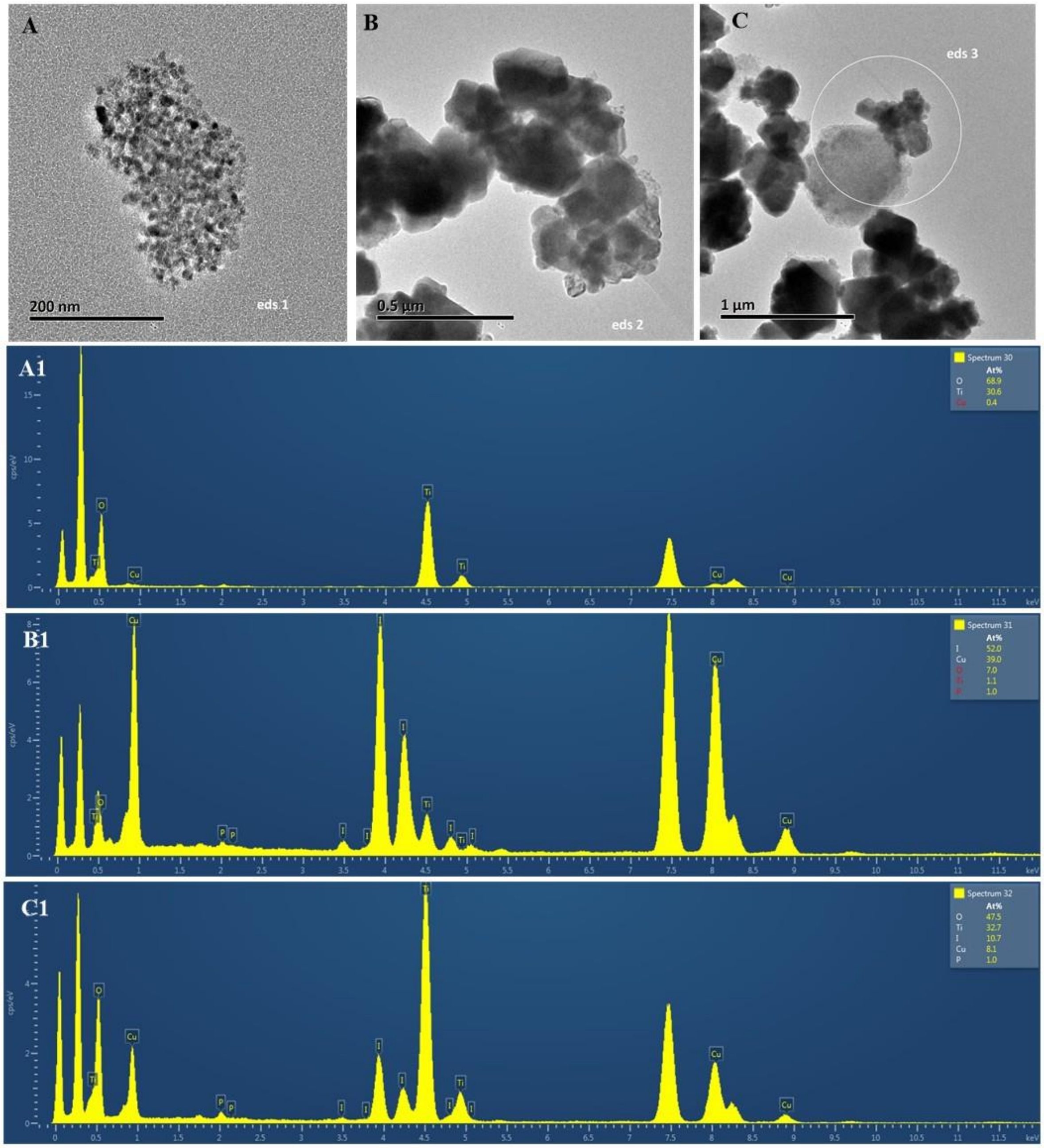

Figure S2. TEM (A, B, and C) and EDX (A1, B1, and C1) analysis of composite (TiO_2-Cu^{2+}/CuI) NMs. TEM micrographs show the presence of crystalline CuI surrounded by TiO_2-Cu^{2+}. EDX (A1, B1, C1) results show the purity and composition of the composite NMs.

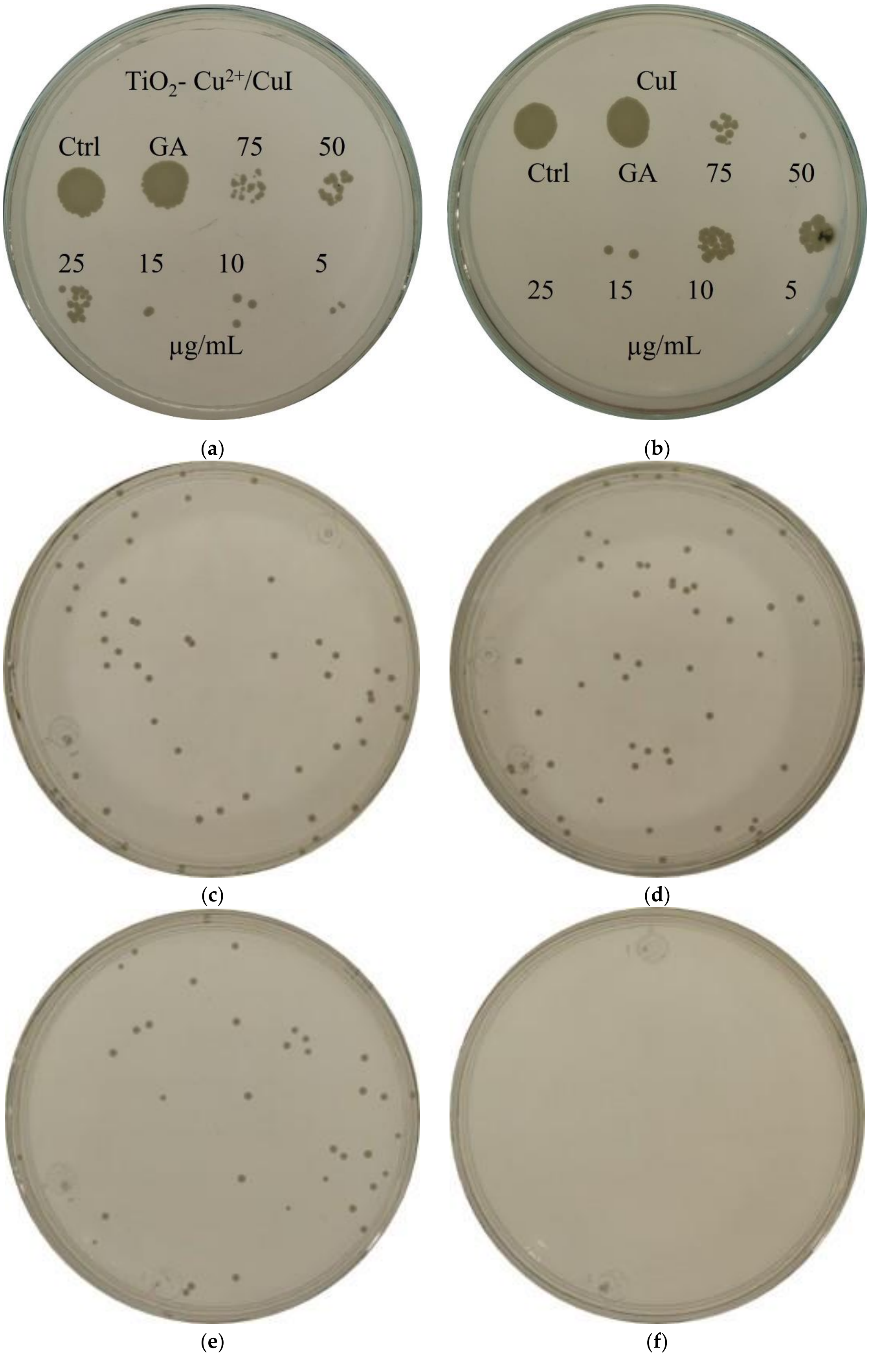
TiO2- Cu2+/CuI
Ctrl GA 75 50
25 15 10 5
μg/mL
CuI
Ctrl GA 75 50
25 15 10 5
μg/mL

(a) (b) (c) (d) (e) (f)

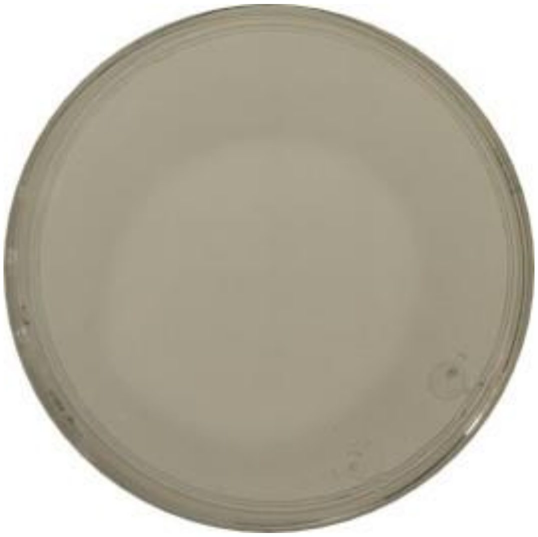

(**g**)

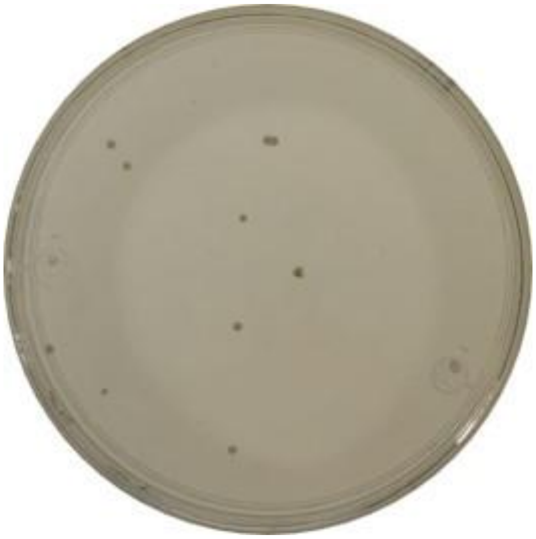

(**h**)

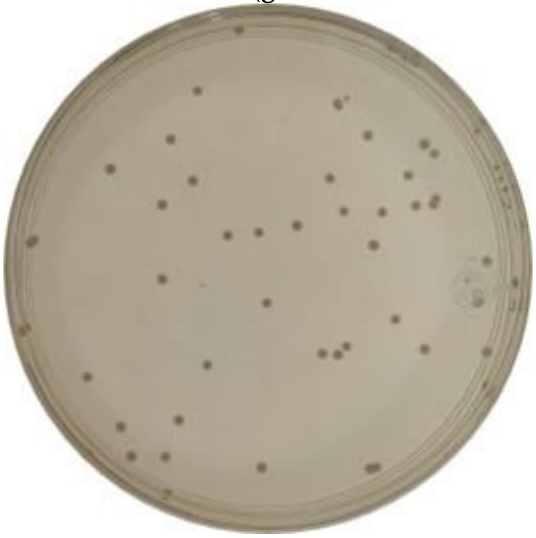

(**i**)

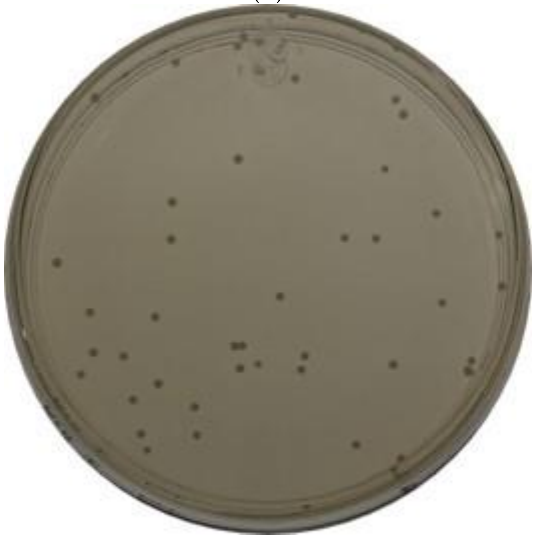

(**j**)

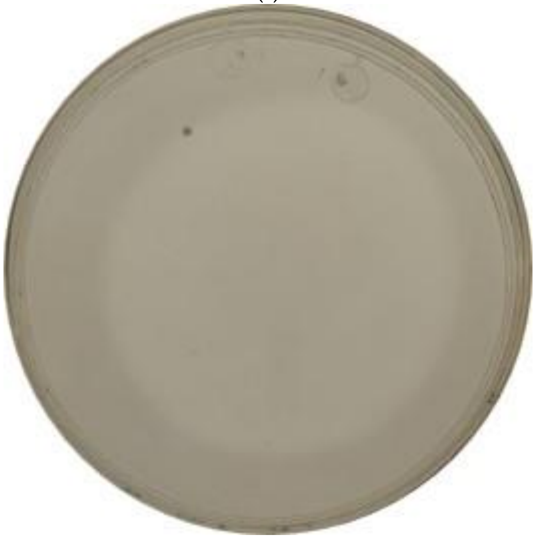

(**k**)

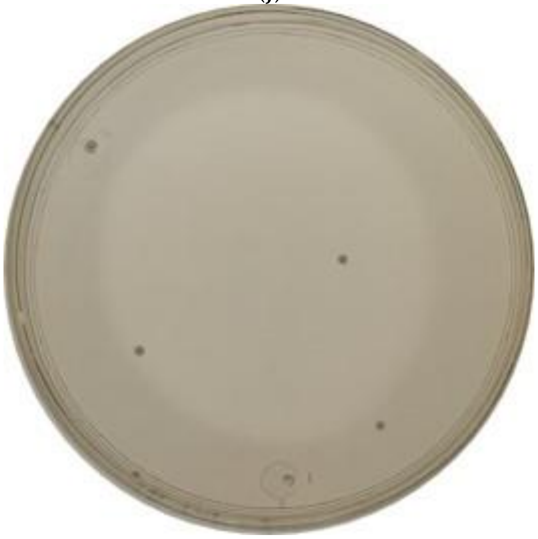

(**l**)

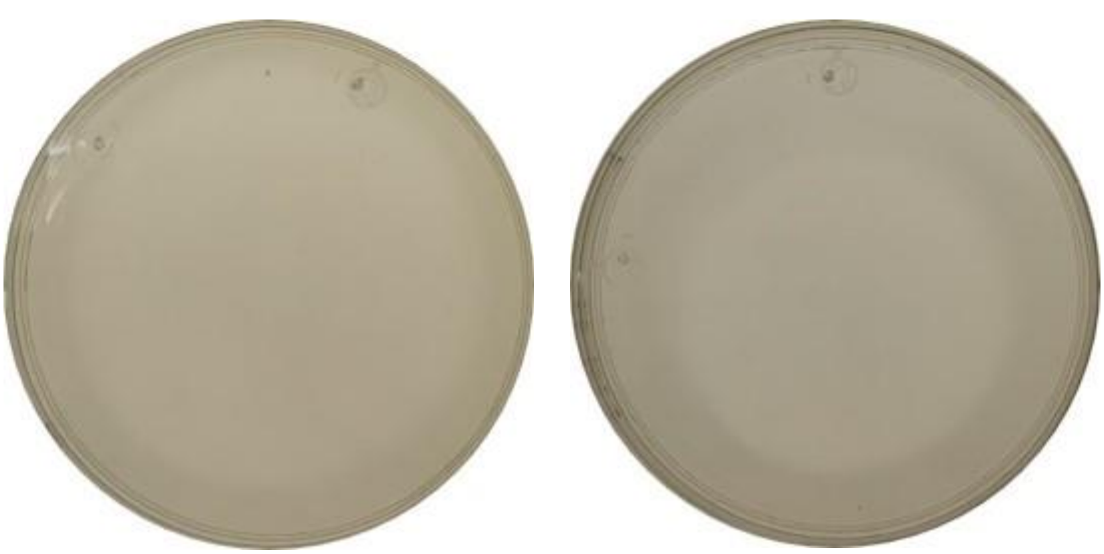

(**m**) (**n**)

Figure S3. *C. parapsilosis* culture on agar after NMs interaction; (**a**) 1h preliminary test with TiO_2-Cu^{2+}/CuI composite; (**b**) 1h preliminary test with CuI; (**c – h**) *C. parapsilosis* vs TiO_2-Cu^{2+}/CuI composite 2 h interaction;(**c**) Control group diluted for counting; (**d**) GA group diluted for counting; (**e**) 5 μg/mL TiO_2-Cu^{2+}/CuI; (**f**) 10 μg/mL TiO_2-Cu^{2+}/CuI; (**g**) 15 μg/mL TiO_2-Cu^{2+}/CuI; (**g**) 25 μg/mL TiO_2-Cu^{2+}/CuI; (**i – n**) *C. parapsilosis* vs CuI NMs 2 h interaction; (**i**) Control group diluted for counting; (**j**) GA group diluted for counting; (**k**) 5 μg/mL CuI; (**l**) 10 μg/mL CuI; (**m**) 15 μg/mL CuI; (**n**) 25 μg/mL CuI.

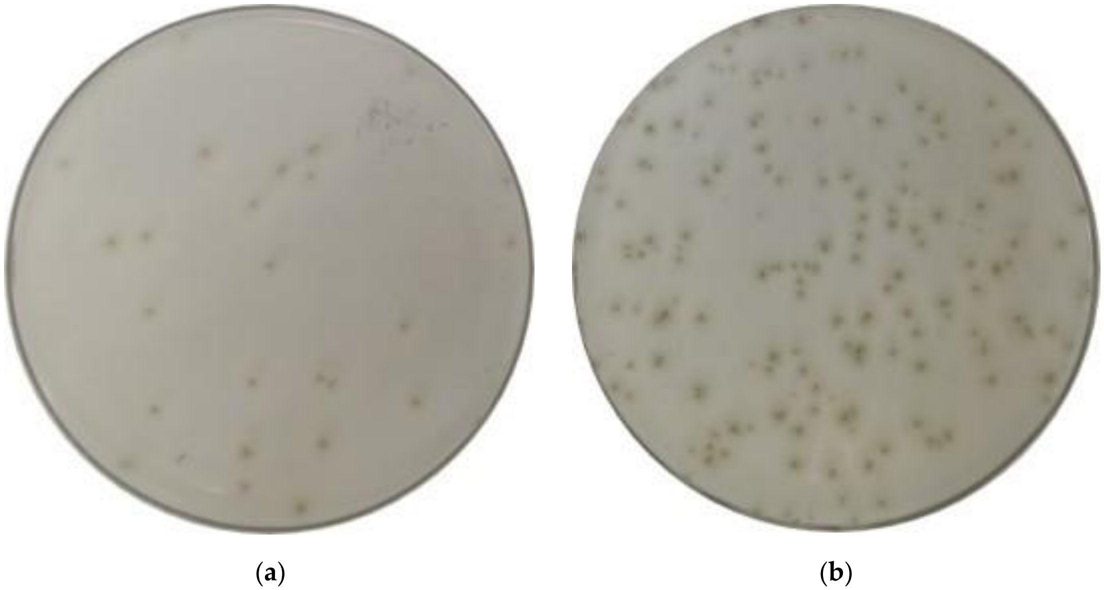

(**a**) (**b**)

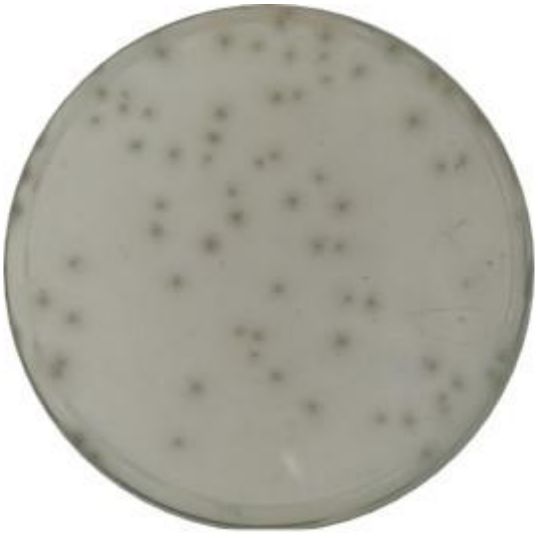

(**c**)

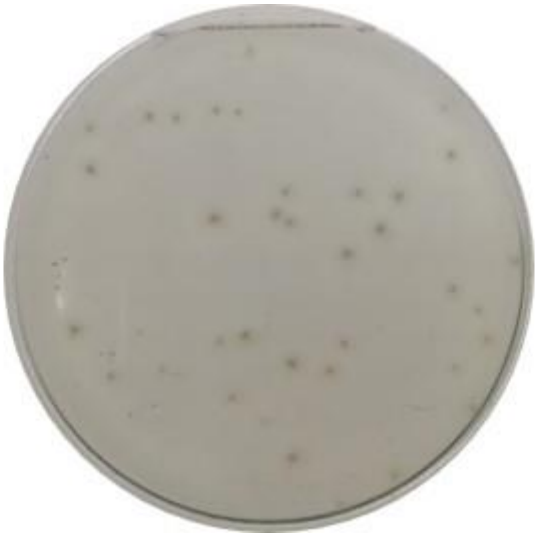

(**d**)

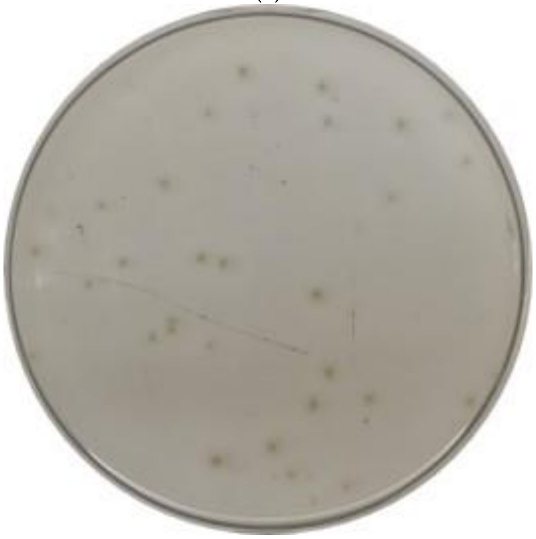

(**e**)

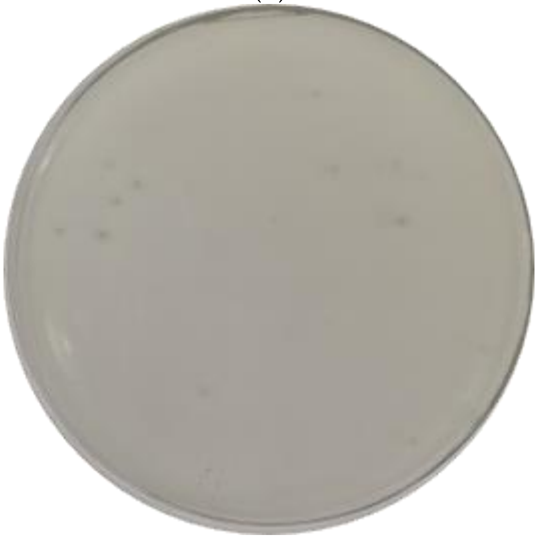

(**f**)

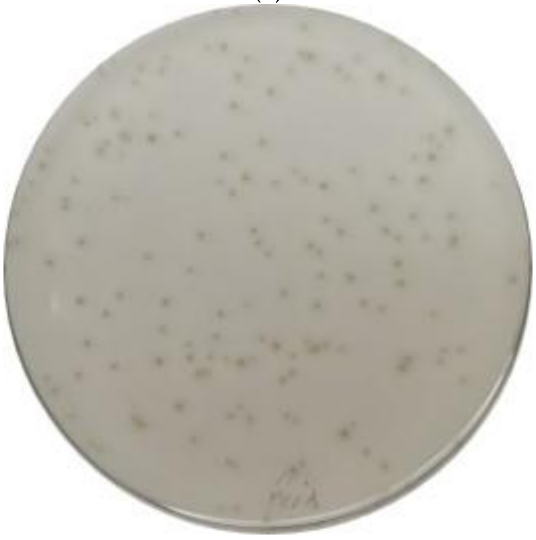

(**g**)

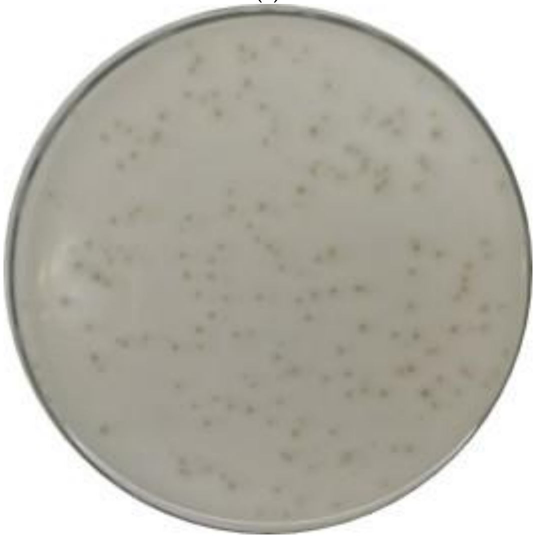

(**h**)

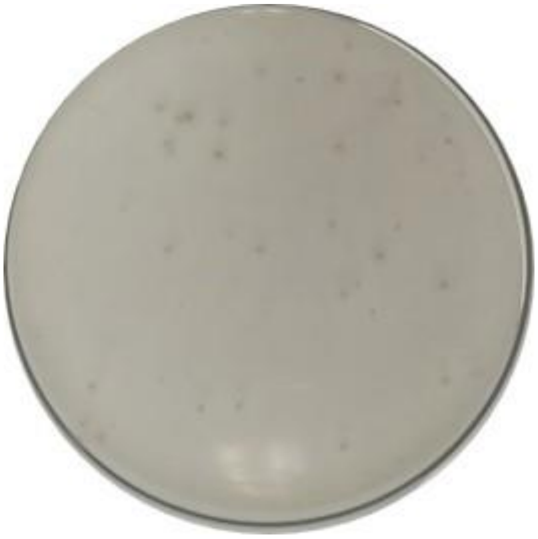

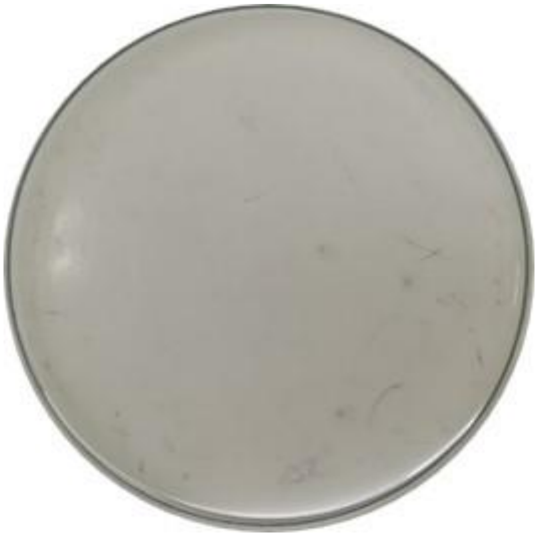

(**i**) (**j**)

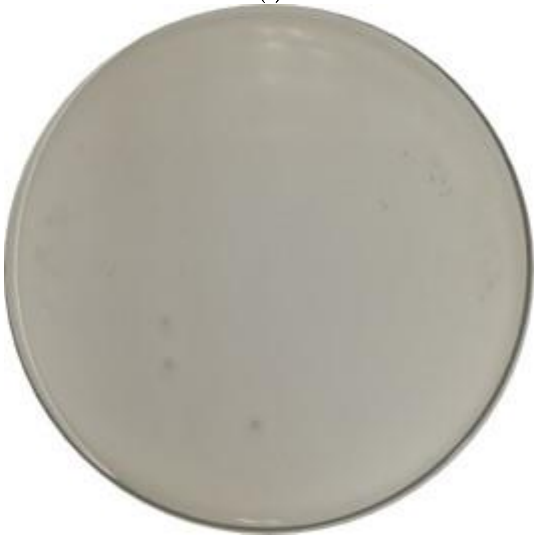

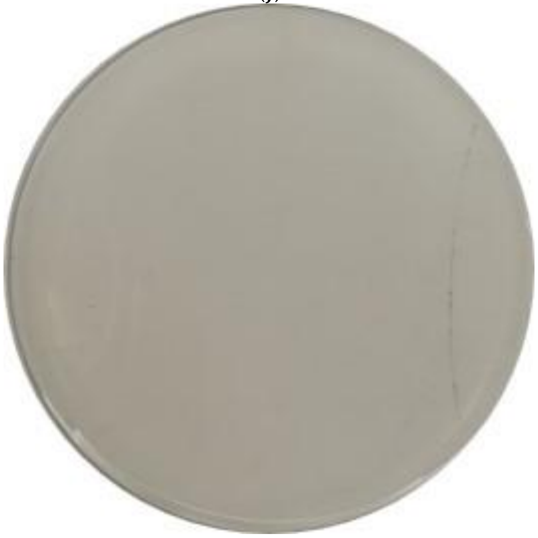

(**k**) (**l**)

Figure S4. *A. niger* culture on agar after NMs interaction; (**a** – **f**) Interaction test series against TiO_2-Cu^{2+}/CuI composite; (**a**) *A. niger* control group diluted for counting; (**b**) 275 μg/mL TiO_2-Cu^{2+}/CuI; (**c**) 350 μg/mL TiO_2-Cu^{2+}/CuI; (**d**) 425 μg/mL TiO_2-Cu^{2+}/CuI; (**e**) 500 μg/mL TiO_2-Cu^{2+}/CuI; (**f**) 575 μg/mL TiO_2-Cu^{2+}/CuI; (**g** – **l**) Interaction test series against CuI; (**g**) *A. niger* control group diluted for counting; (**h**) 275 μg/mL CuI; (**i**) 350 μg/mL CuI; (**j**) 425 μg/mL CuI; (**k**) 500 μg/mL CuI; (**l**) 575 μg/mL CuI.

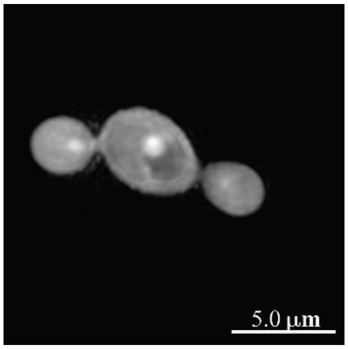

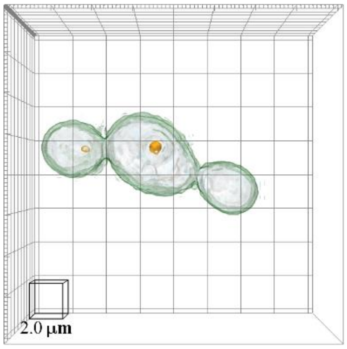

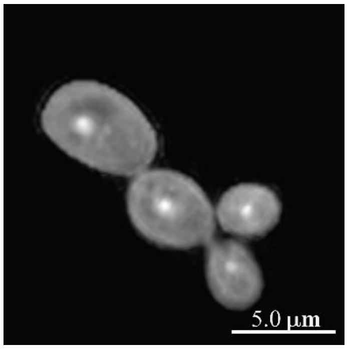

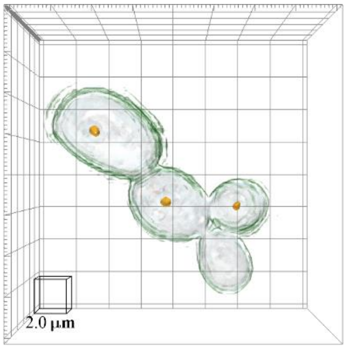

(**a**) (**b**) (**c**) (**d**)

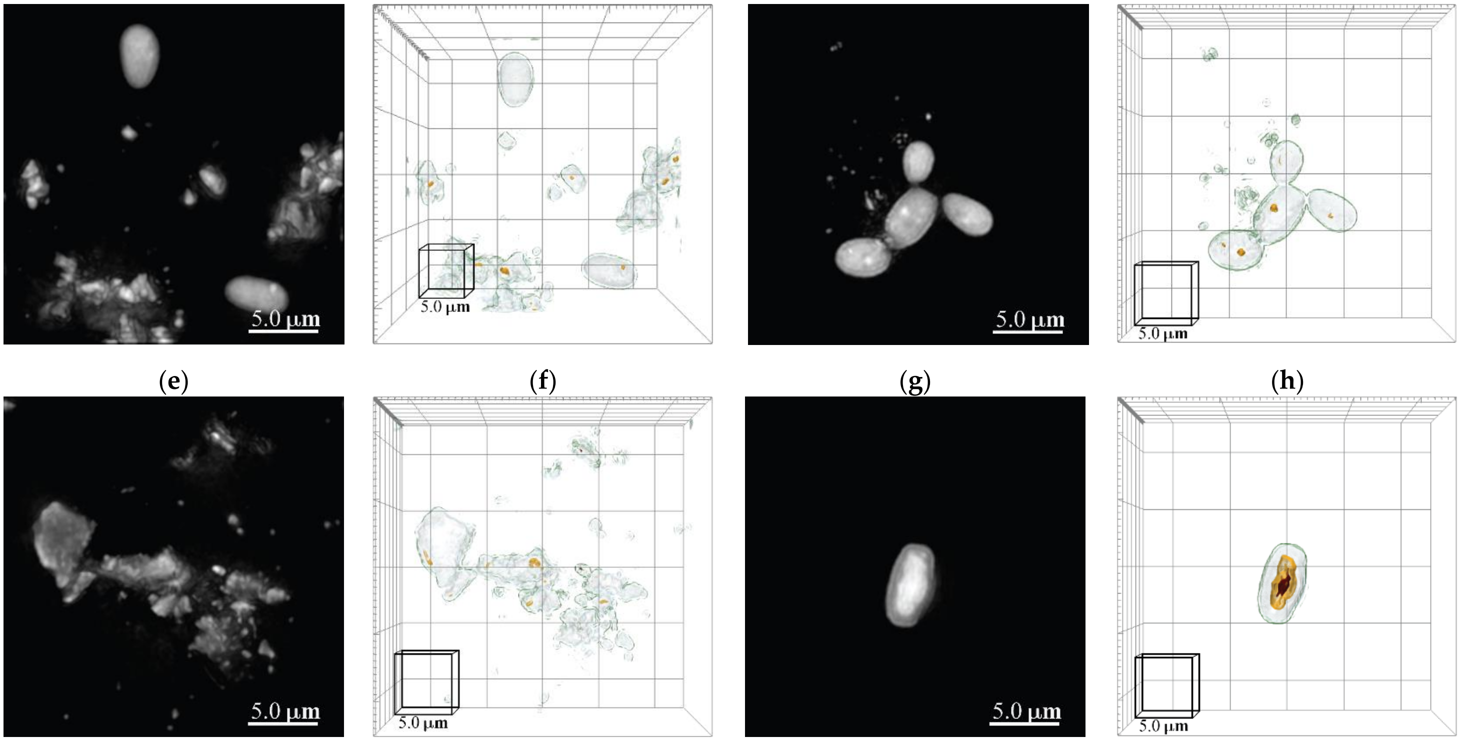

(e) (f) (g) (h)

(i) (j) (k) (l)

Figure S5. HT microscopy analysis of *C. parapsilosis* – NMs interaction: (**a – d**) RI tomogram, 3D reconstruction and bright field images of *C. parapsilosis* control group; (**d, e, f**) RI tomogram, 3D reconstruction and bright field images of *C. parapsilosis* – CuI interacion; (**g, h, i**) RI tomogram, 3D reconstruction and bright field images of *C. parapsilosis* – TiO_2-Cu^{2+}/CuI interacion.

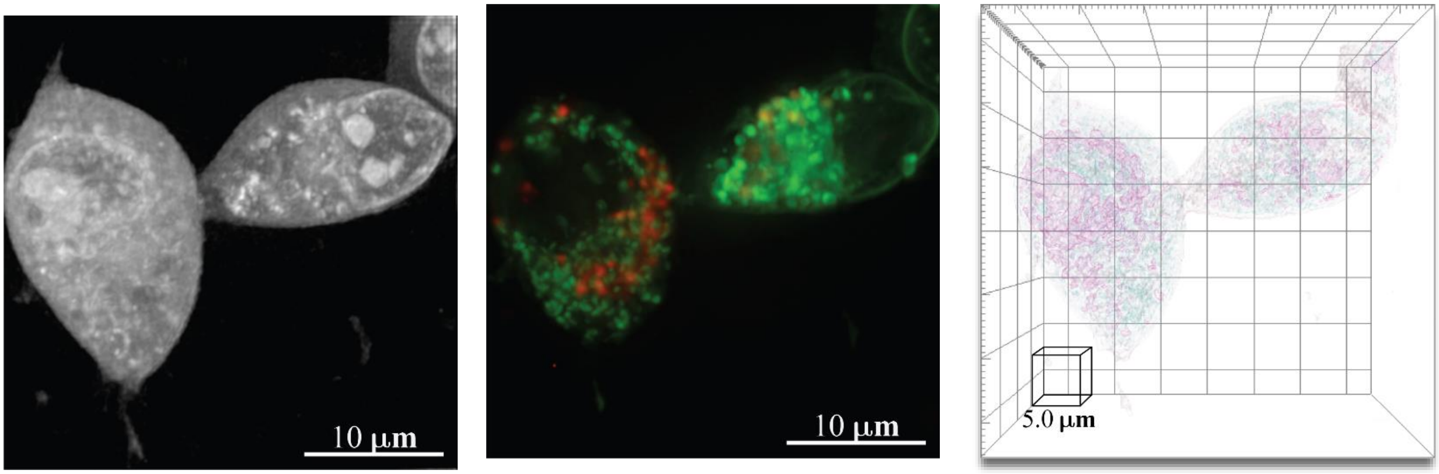

(**a**) (**b**) (**c**)

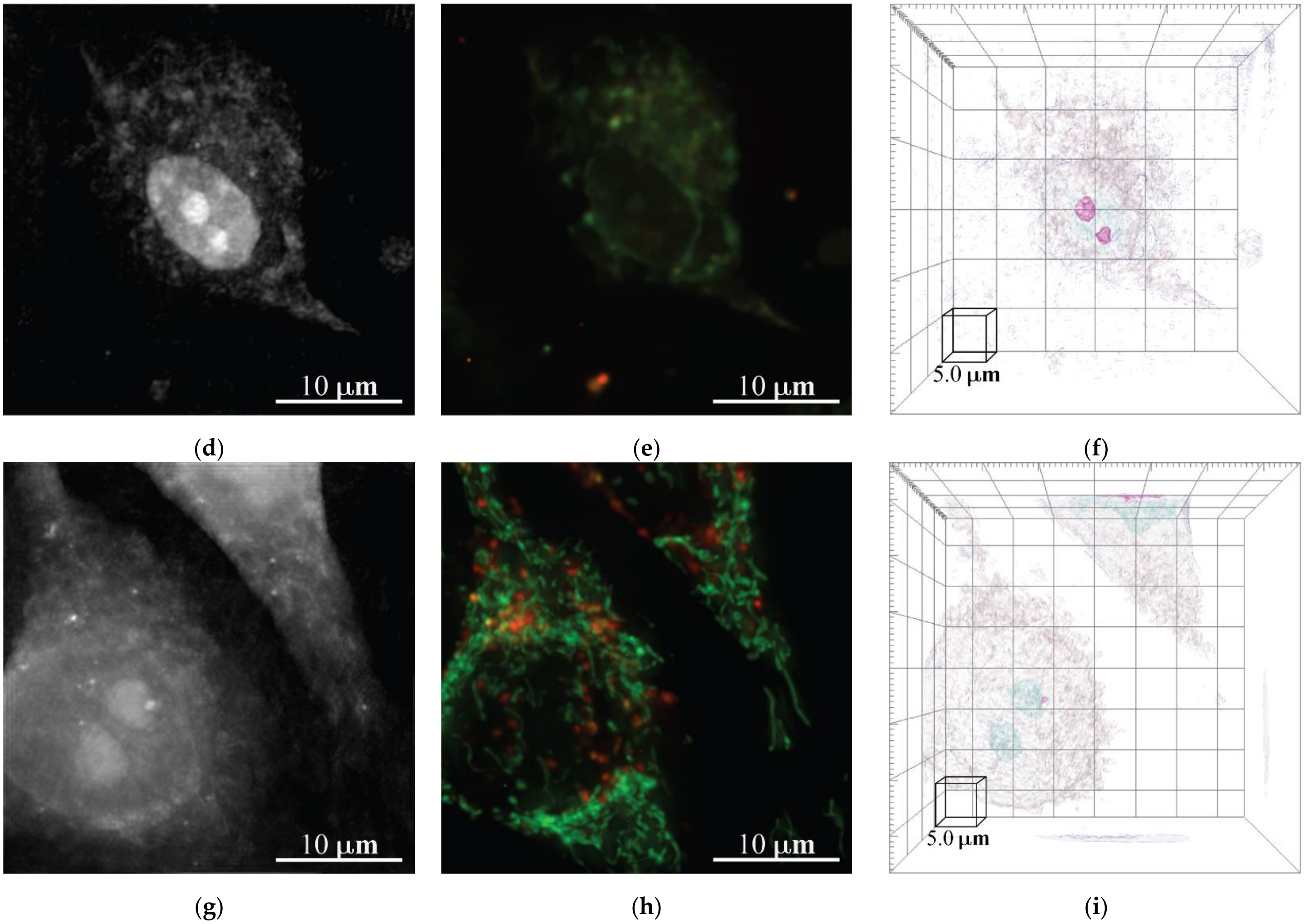

Figure S6. HT microscopy evaluation of BEAS 2B – CuI and TiO_2-Cu^{2+}/CuI NMs interaction: RI tomogram, fluorescence marker and 3D reconstruction of: (**a** – **c**) BEAS 2B control group; (**d** – **f**) BEAS 2B – CuI interaction; (**g** – **h**) BEAS 2B – TiO_2-Cu^{2+}/CuI interaction.

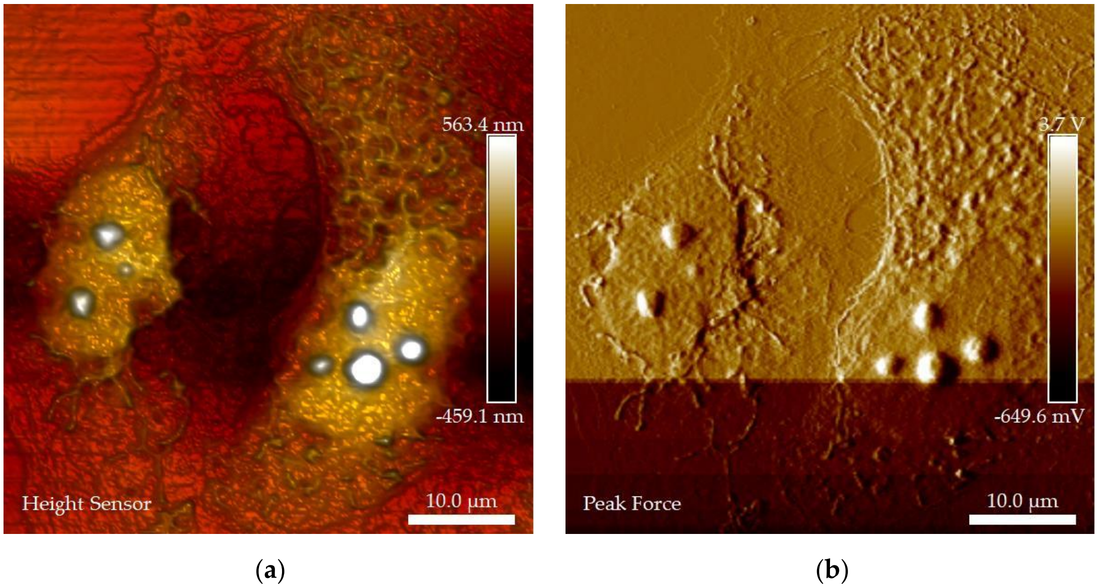

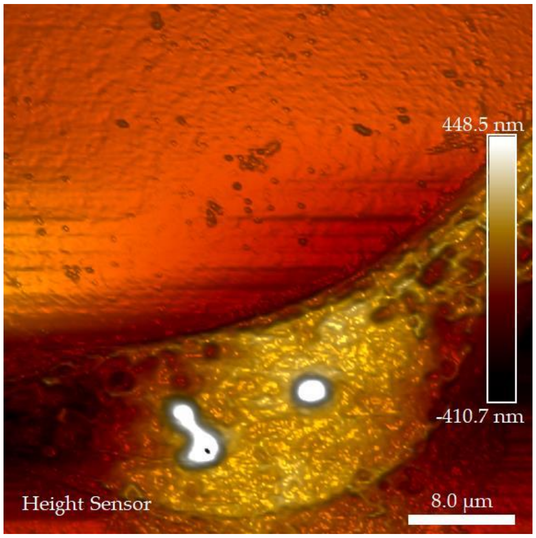

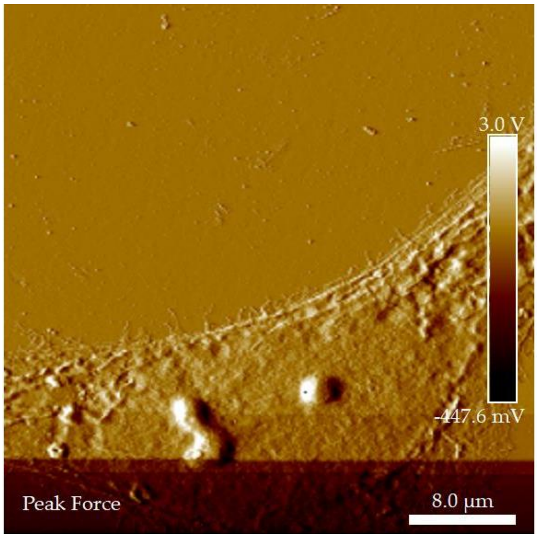

(**c**) (**d**)

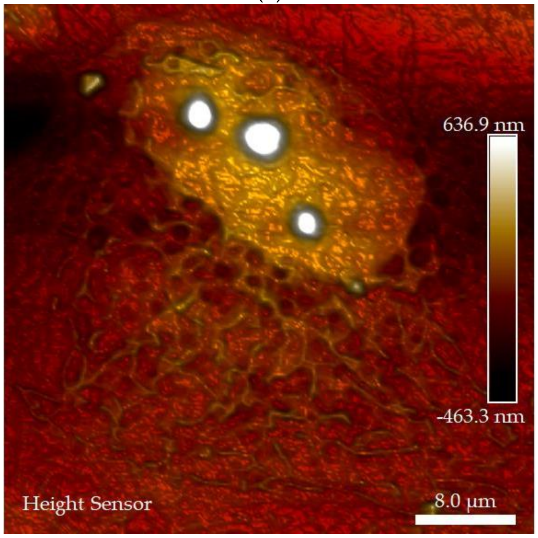

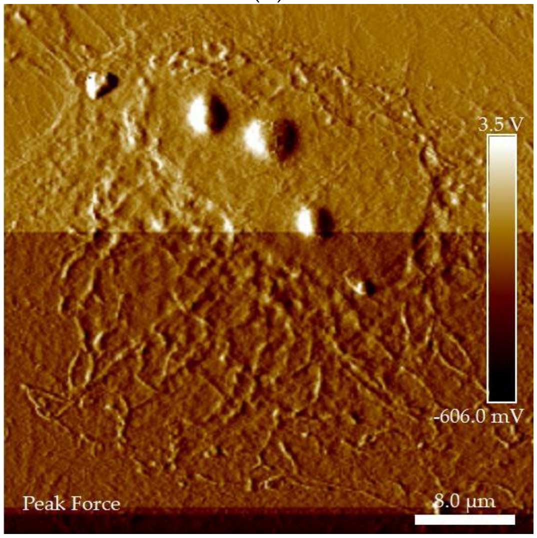

(**e**) (**f**)

Figure S7. AFM microscopy evaluation of BEAS 2B control group: (**a, c, e**) BEAS 2B height sensor; (**b, d, f**) BEAS 2B peak force.

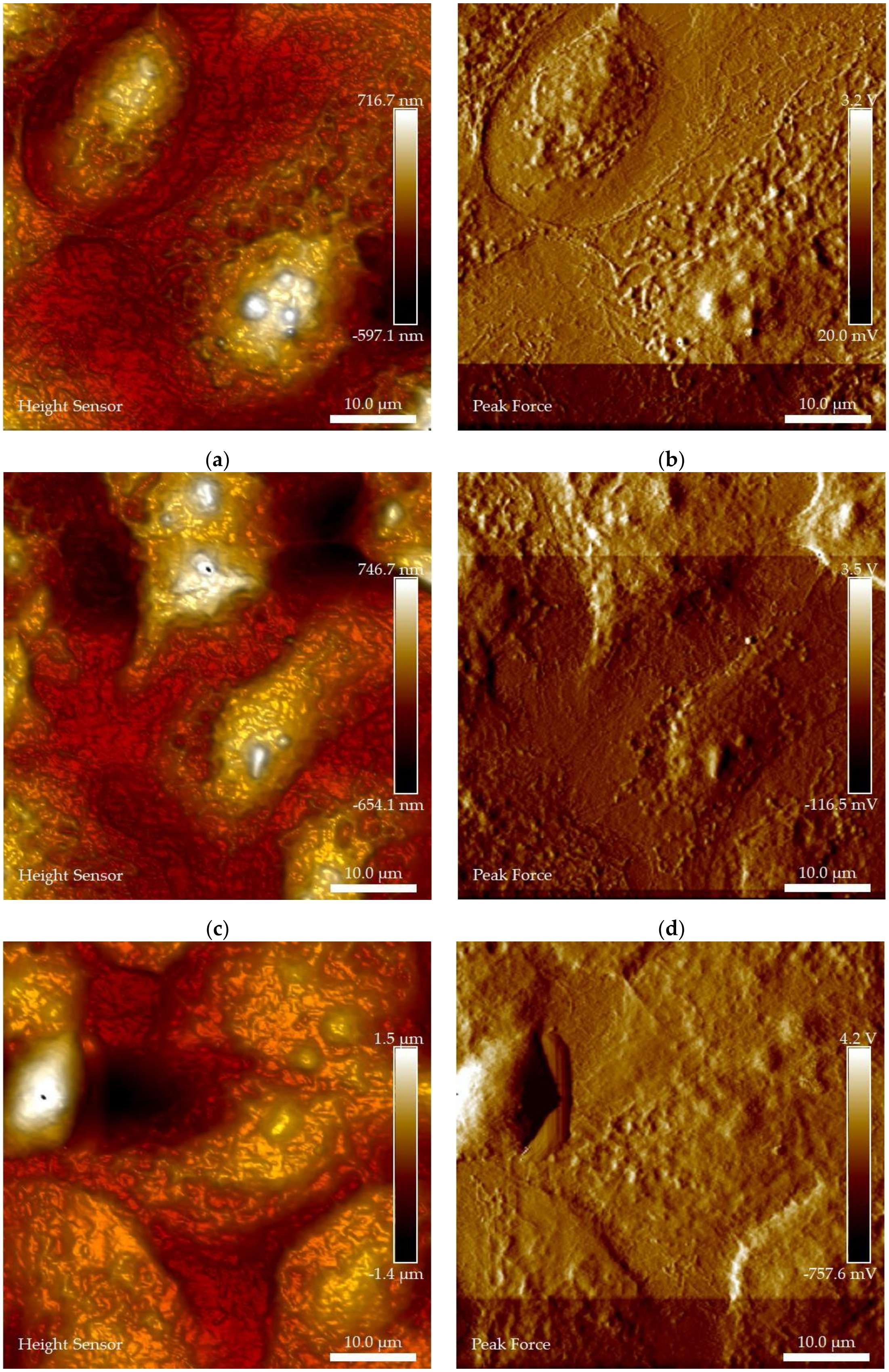

716.7 nm
-597.1 nm
Height Sensor
10.0 µm
3.2 V
20.0 mV
Peak Force
10.0 µm
(a)
(b)
746.7 nm
-654.1 nm
Height Sensor
10.0 µm
3.5 V
-116.5 mV
Peak Force
10.0 µm
(c)
(d)
1.5 µm
-1.4 µm
Height Sensor
10.0 µm
4.2 V
-757.6 mV
Peak Force
10.0 µm

(**e**) (**f**)

Figure S8. AFM microscopy evaluation of BEAS 2B – CuI interaction test: (**a, c, e**) BEAS 2B height sensor; (**b, d, f**) BEAS 2B peak force.

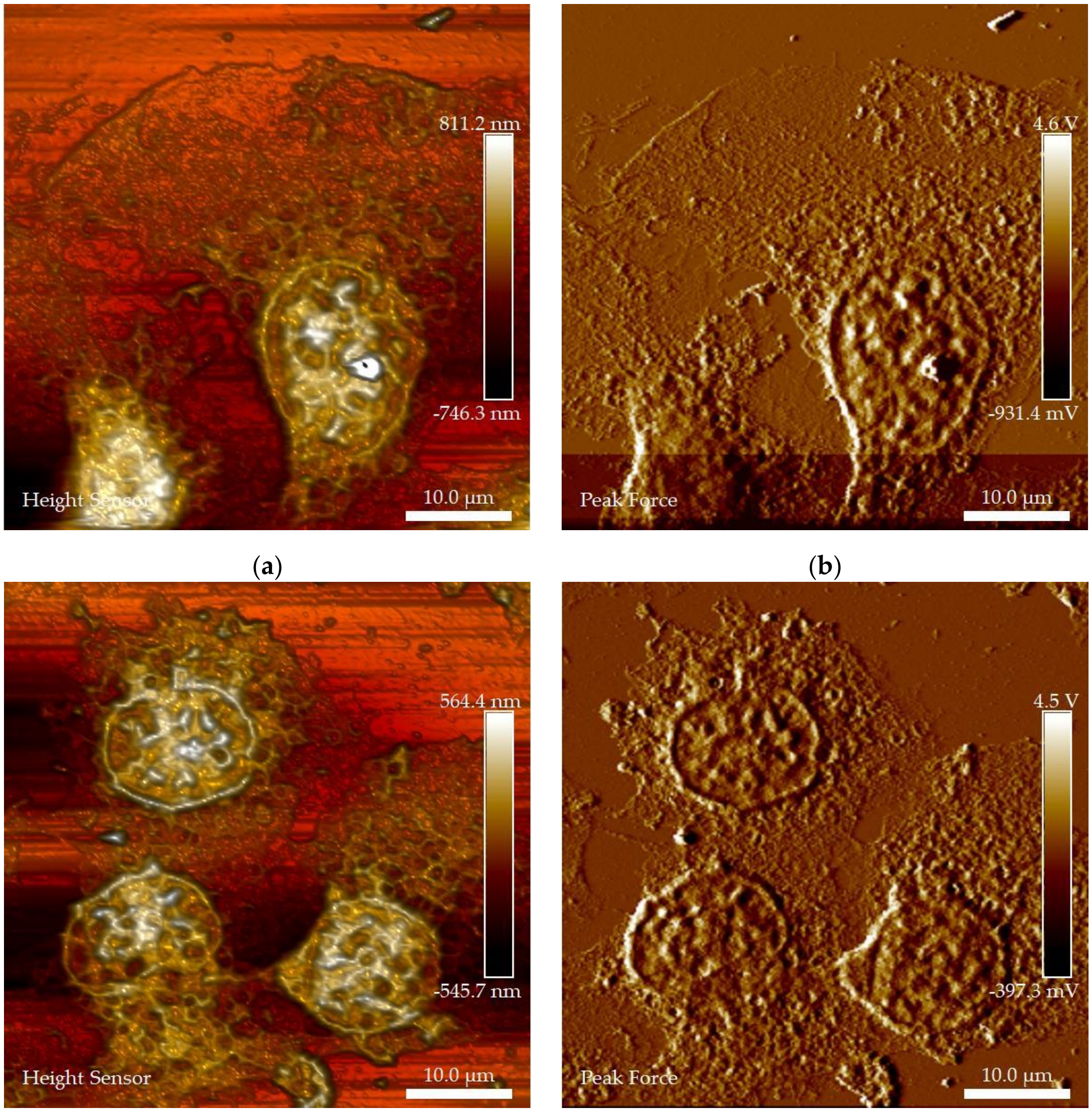

(**a**) (**b**)

(**c**) (**d**)

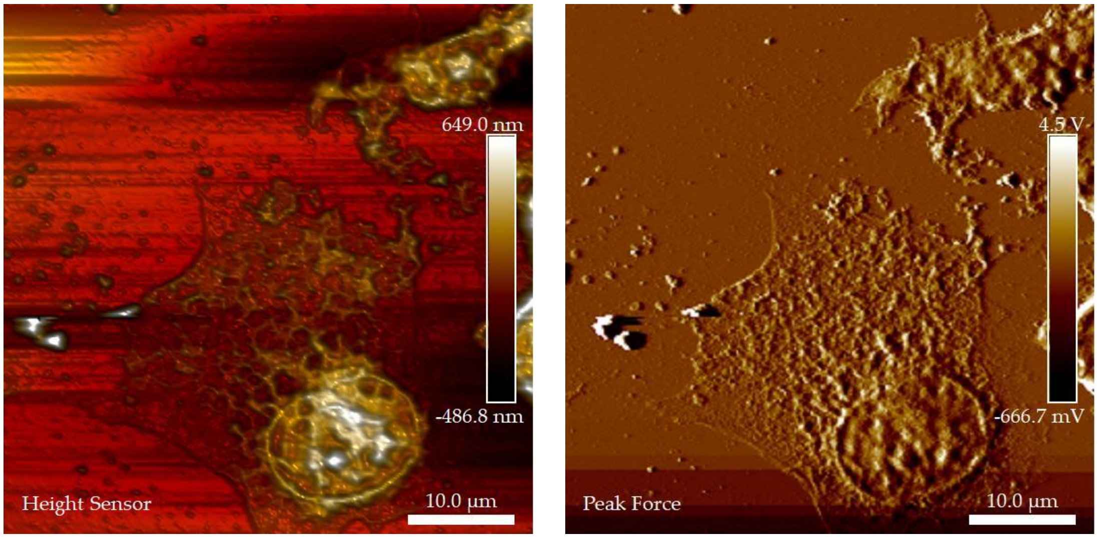

(**e**) (**f**)

Figure S9. AFM microscopy evaluation of BEAS 2B – TiO_2-Cu^{2+}/CuI interaction test: (**a, c, e**) BEAS 2B height sensor; (**b, d, f**) BEAS 2B peak force.
